# Oxygen Sensing Mesenchymal Progenitors Promote Neo-Vasculogenesis in a Humanized Mouse Model *In Vivo*

**DOI:** 10.1371/journal.pone.0044468

**Published:** 2012-09-07

**Authors:** Nicole A. Hofmann, Anna Ortner, Rodrigo O. Jacamo, Andreas Reinisch, Katharina Schallmoser, Rokhsareh Rohban, Nathalie Etchart, Margareta Fruehwirth, Christine Beham-Schmid, Michael Andreeff, Dirk Strunk

**Affiliations:** 1 Stem Cell Research Unit, Medical University of Graz, Graz, Austria; 2 Department of Leukemia, M.D. Anderson Cancer Center, University of Texas, Houston, Texas, United States of America; 3 Department of Hematology and Stem Cell Transplantation, Medical University of Graz, Graz, Austria; 4 Department of Blood Group Serology and Transfusion Medicine, Medical University of Graz, Graz, Austria; 5 Institute of Pathology, Medical University of Graz, Graz, Austria; University of Cincinnati, United States of America

## Abstract

Despite insights into the molecular pathways regulating hypoxia-induced gene expression, it is not known which cell types accomplish oxygen sensing during neo-vasculogenesis. We have developed a humanized mouse model of endothelial and mesenchymal progenitor co-transplantation to delineate the cellular compartments responsible for hypoxia response during vasculogenesis. Mesenchymal stem/progenitor cells (MSPCs) accumulated nuclear hypoxia-inducible transcription factor (HIF)-1α earlier and more sensitively than endothelial colony forming progenitor cells (ECFCs) *in vitro* and *in vivo*. Hypoxic ECFCs showed reduced function *in vitro* and underwent apoptosis within 24h *in vivo* when used without MSPCs. Surprisingly, only in MSPCs did pharmacologic or genetic inhibition of HIF-1α abrogate neo-vasculogenesis. HIF deletion in ECFCs caused no effect. ECFCs could be rescued from hypoxia-induced apoptosis by HIF-competent MSPCs resulting in the formation of patent perfused human vessels. Several angiogenic factors need to act in concert to partially substitute mesenchymal HIF-deficiency. Results demonstrate that ECFCs require HIF-competent vessel wall progenitors to initiate vasculogenesis *in vivo* and to bypass hypoxia-induced apoptosis. We describe a novel mechanistic role of MSPCs as oxygen sensors promoting vasculogenesis thus underscoring their importance for the development of advanced cellular therapies.

## Introduction

Vascular homeostasis and regeneration play an essential role in development, health and disease [1], [2]. Vessel remodeling and repair during postnatal life have long been viewed as occurring exclusively through proliferation and subsequent migration of mature ECs derived from pre-existing vessel walls, a process termed angiogenesis [3]. The isolation of EC progenitors from human blood changed that paradigm and introduced the concept of therapeutic vasculogenesis [4]. The discovery that vessel wall-derived ECs rapidly proliferate because they contain a complete hierarchy of ECFCs supported the concept of the progenitor-dependence of vasculogenesis [5]–[8]. Cell transplantation to re-vascularize ischemic tissue has thus become a central vision for regenerative medicine [8]–[10]. Therapeutic targets comprise cardiovascular diseases including stroke, myocardial infarction and peripheral artery disease as well as wound healing and vessel creation as the prerequisite for effective tissue engineering [10].

Initial attempts towards vascular regenerative cell therapy used sole EC or progenitor transplantation. More stable vessel formation during adult neo-vasculogenesis was achieved by the co-transplantation of ECs with stromal cells or more complex progenitor transplants [11]–[14]. The robust vasculogenic program that endothelial and mesenchymal progenitors can preserve despite *ex vivo* propagation is also evidenced by the fact that the progeny of expanded hemangioma-derived stem cells can still mimic disease pathology after xeno-transplantation [15]. Different types of stromal cells have since been shown to assume mural cell function stabilizing human vessels after co-transplantation in several mouse models [12], [14], [16]–[18]. However, the effectors initiating neo-vasculogenesis are as yet unknown.

Most of our current insights into the molecular control of vessel formation have been derived from studying sprouting angiogenesis [10], [19]. This process involves HIF-1α which is constitutively expressed and instantaneously degraded at sufficient oxygen tension. Under hypoxia, HIF-1α is stabilized and acts together with HIF-1β as a transcription factor for a multiplicity of target genes carrying hypoxia response elements [20], [21]. The current view is that stabilized HIF drives an angiogenic program that results in EC sprouting from existing vessels. The hypoxia-induced production of growth factors is considered to subsequently recruit mesenchymal cells to adapt a pericyte phenotype providing structural stability of the newly formed vessels [1], [10], [22]. It is not clear whether the same sequence of events is operative during progenitor-derived adult vasculogenesis. Therapeutic vasculogenesis has so far remained an unmet medical need at least in part due to our limited understanding of the interplay between endothelial and mesenchymal progenitors during re-vascularization of ischemic tissue [9], [10]. Here we show for the first time that MSPCs act by sensing low oxygen thereby enabling the initiation of neo-vasculogenesis in addition to their established function as vessel stabilizing pericytes.

**Figure 1:**
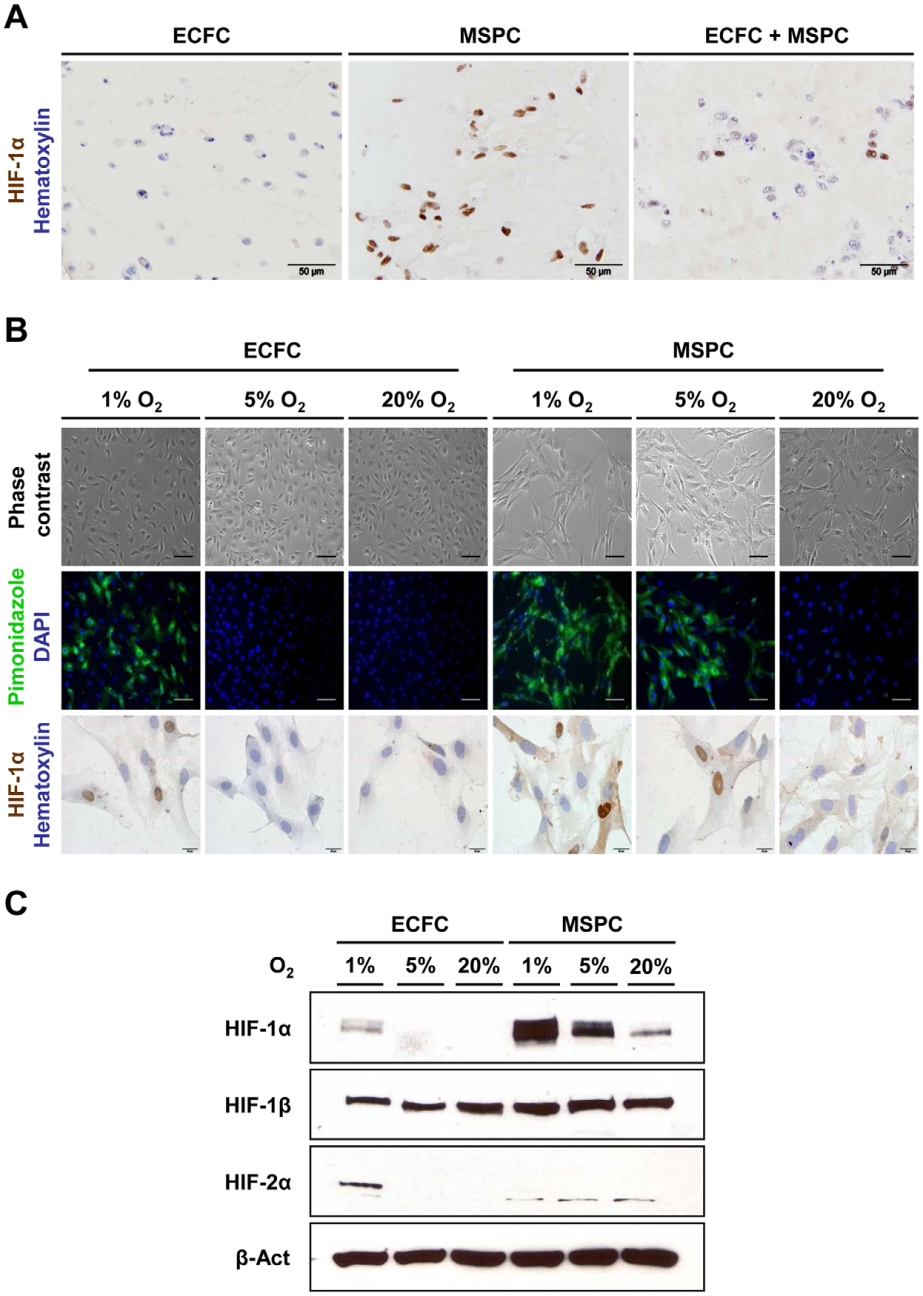
Mural cells are more sensitive to hypoxia than endothelial cells. (**A**) Immune histochemical staining of Matrigel plugs 1 day (d) after implantation of either ECFCs only (left picture), ECFCs/MSPCs together (middle picture) or MSPCs only (right picture). Plugs were explanted and sections were stained with anti-HIF-1α (DAB, brown) and co-stained with hematoxylin (blue; scale bar 50 µm). Staining revealed that 100% of MSPCs display positive signals for HIF-1α, while ECFCs alone show no HIF-1α stabilization and in plugs of ECFCs together with MSPCs the number of positive cells corresponds to the 20% MSPCs. (**B**) To measure the hypoxia response at the single cell level *in vitro*, ECFCs and MSPCs were cultured for 4 h at 1%, 5% and 20% O_2_. Phase contrast microphotographs show an unchanged appearance. Hypoxyprobe (pimonidazole, green; 4′,6-Diamidin-2-phenylindol, DAPI nuclear stain in blue) revealed that MSPCs sense hypoxia at 5% O_2_ and both ECFCs and MSPCs sense hypoxia at 1% O_2_. Accordingly, nuclear HIF-1α protein accumulation was detected in MSPCs at 5% and in both progenitor cell types at 1% O_2_ (scale bar 100 µm for phase contrast and pimonidazole and 20 µm for HIF-1α pictures). (**C**) Equal amounts of protein from cell lysates corresponding to the analysis in (B) were tested in western blots showing higher amounts of HIF-1α in MSPCs. MSPCs stabilize HIF-1α at 5% and display a minute baseline signal at ambient air levels of 20% O_2_ (n = 3; representative blot regions are shown; see Figure S1E for complete blot scans).

## Methods

### Ethics Statement

Prior approval was obtained for human cell and tissue sample collection from the Institutional Review Board of the Medical University of Graz (protocols 19-252 ex 07/08, 18-243 ex 06/07, 21.060 ex 09/10). Adult samples were collected after written informed consent from healthy volunteers and cardiovascular disease (CVD) patients, and umbilical cord or cord blood samples after written informed consent by the mother after full-term pregnancies in accordance with the Declaration of Helsinki. Animal experiments were approved by the Animal Care and Use Committee at the Veterinary University of Vienna on behalf of the Austrian Ministry of Science and Research according to the criteria published in the guide for the care and use of laboratory animals and performed as described [17]. Neonatal fibroblasts were purchased from Lonza (Walkersville, MD).

**Figure 2:**
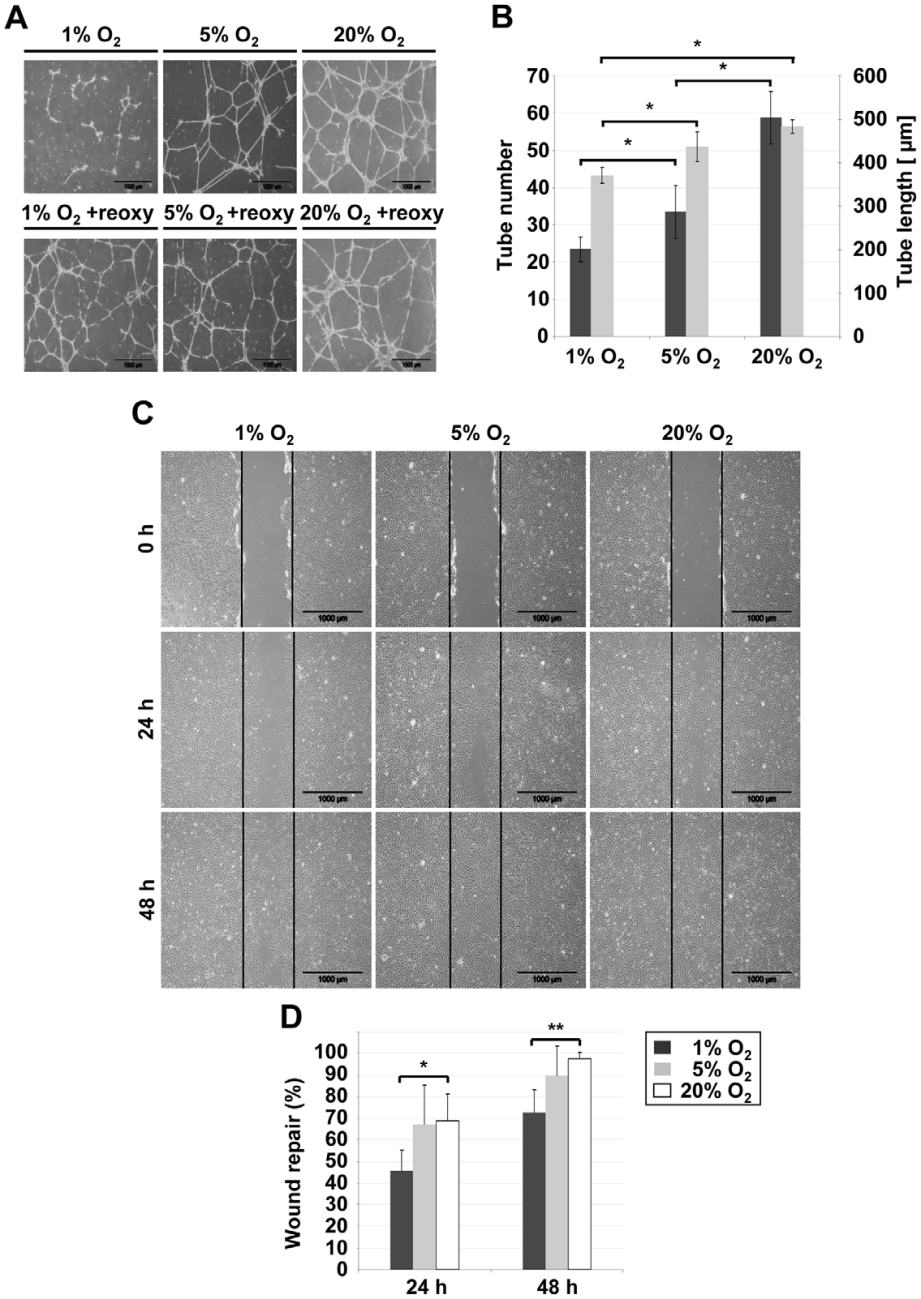
Functional quiescence of progenitor cells under hypoxia *in vitro*. (**A**) Vascular-like structures documented 24 h after seeding 1.8×10^5^ ECFCs per 10 cm^2^ on top of a matrix gel in a standard angiogenesis assay showing impaired network formation by ECFCs at 1% more than at 5% O_2_ (upper row) and resumed vessel-like structure formation after re-oxygenation back to 20% O_2_ standard conditions (+reoxy, lower row; one representative picture series; n = 3). (**B**) Tube number (black bar) and length (grey bar) as determined using ImageJ software (http://rsbweb.nih.gov) were significantly reduced with decreasing O_2_ (mean ± SD; *p<0.05; n = 4). (**C**) Wounding an ECFC-derived monolayer in a scratch assay was used to monitor endothelial wound repair under hypoxia (1% O2) as compared to reduced (5% O_2_) and ambient air (20% O_2_) standard laboratory test conditions (representative examples are shown; see also Video S1). (**D**) A significantly decreased capacity to close an endothelial wound area over time was found at 1% O_2_ compared to 20% O_2_ (n = 5; *p<0.05, **p<0.001).

### Human Progenitor Cell Isolation, Large-scale Expansion and Long-term Propagation

ECFCs and MSPCs were isolated and expanded from neonatal cord or cord blood (CB), adult peripheral blood (PB) or bone marrow (BM) aspirates following IRB approval as previously described under animal serum-free culture conditions with pooled human platelet lysate (pHPL) replacing fetal bovine serum (FBS) [17], [23]–[25]. Additional video protocols covering production of pHPL (www.jove.com/details.php?id=1523) [26], as well as propagation of blood-derived ECFCs (www.jove.com/details.php?id=1524) [27] and umbilical cord ECFCs and MSPCs (www.jove.com/details.php?id=1525) [28] are available online to support reproducibility. For large-scale propagation, primary culture-derived ECFCs were seeded in EGM-2 (Lonza) supplemented with 10% pHPL at a density of 100 cells/cm^2^ and MSPCs in α-MEM (Sigma-Aldrich, St. Louis, MO) also supplemented with 10% pHPL at a density of 30 cells/cm^2^ in 2,528 cm^2^ cell factories (Thermo Fisher Scientific, Freemont, CA). For long-term propagation primary culture-derived ECFCs and MSPCs were seeded in triplicate in 75cm^2^ flasks at a density of 100 cells/cm^2^ (n = 4) and 30 cells/cm^2^ (n = 2), respectively, as described [29]. Cells were cryopreserved after expansion until use as described [30].

**Figure 3:**
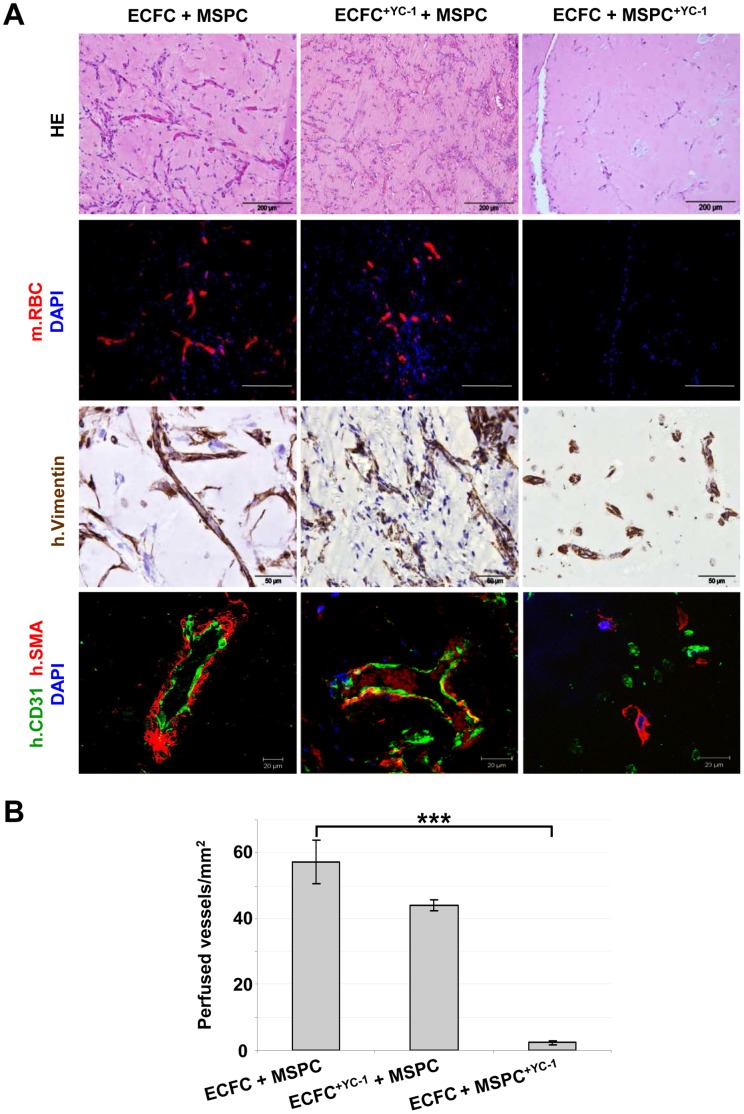
Human vessel formation after progenitor co-transplantation depends on hypoxia-induced factors in MSPCs but not ECFCs. (**A**) Vessel formation was determined 2 weeks after ECFC+MSPC (2×10^6^ cells/300µL, ratio of 80∶20) co-transplantation. Pre-treatment of one of the two co-transplanted cell populations with YC-1 (1 h, 37°C; ECFC^+YC1^+MSPC, n = 3; ECFC + MSPC^+YC1^, n = 5) compared to untreated transplants (ECFC+MSPC, n = 8) as indicated above each column. Hematoxylin/eosin (HE) staining visualizes morphology (first row; scale bar 200 µm) and the mouse red blood cells (mRBC) resulting from perfusion after connection to the recipients’ circulation (Ter119 anti- mouse glycophorin reactivity, red; 4′,6-Diamidin-2-phenylindol, DAPI, blue; second row; scale bar 200 µm). Human mesodermal origin was visualized with anti-human vimentin monoclonal antibody staining (h.Vimentin, brown; mouse and human nuclei counterstained with hematoxylin, blue; third row; scale bar 50 µm). Plugs contain different numbers of h.Vimentin-negative infiltrating mouse CD45^+^ hematopoietic cells (see Figure S6C). Fourth row indicating presence or absence of human vessels stained with rabbit anti-human CD31 labeling ECFCs (h.CD31, green), mouse anti-human alpha smooth muscle actin labeling pericytes and mural smooth muscle cells (h.SMA, red) and nuclear counter-stain (DAPI, blue; scale bar 20 µm). (**B**) Microvessel density was quantified in 200x magnifications of HE stained Matrigel plug sections by ImageJ and counting red blood cell-filled vessel structures. A significantly decreased capacity to form perfused vessels was found in implants containing YC-1 pre-treated MSPCs compared to untreated co-transplants. (n = 3, 5 high power fields 200x; ***p<0.0001).

**Figure 4:**
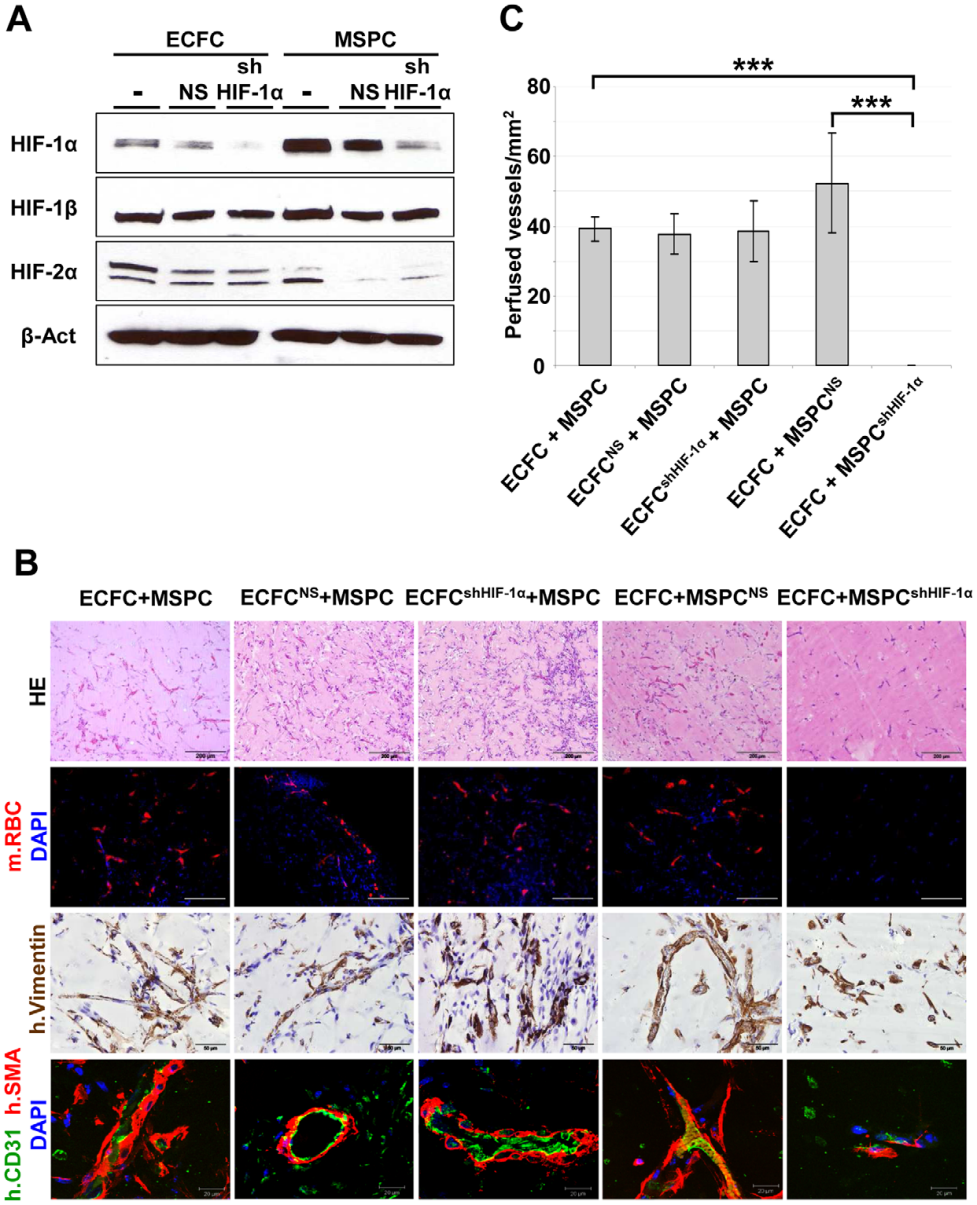
Specific knock-down of HIF-1α in MSPCs but not in ECFCs inhibits vessel formation *in vivo*. (**A**) Total cell lysates of untreated control (**−**) ECFCs and MSPCs or after infection with either pGIPZ-HIF1α-shRNA (shHIF-1α) or non-specific pGIPZ-scramble-shRNA (NS) were separated by SDS-PAGE after 6 hours of incubation at 1% O_2_. Blots were stained with HIF-1α, HIF-2α, HIF-1β or β-actin (β-Act). Full blots are shown in Figure S2. (**B**) To delineate the impact of genetic ablation of HIF-1α in either ECFCs or MSPCs before co-transplantation, vessel formation was determined one week after co-transplantation of HIF-1α-silenced (ECFC^shHIF-1α^, n = 3; MSPC^shHIF-1α^, n = 7) or mock-transfected non-silenced cells (ECFC^NS^, n = 3; MSPC^NS^, n = 7) with genetically un-manipulated corresponding (+ECFC and +MSPC) partner cells as indicated above the columns. Analysis was performed as specified in Figure 3A and is identified at the left side of the picture. Scale bars indicate magnification (first and second row, 200 µm; third row, 50 µm; fourth row, 20 µm). (**C**) Microvessel density was quantified in 200x magnifications of HE stained Matrigel plug sections by ImageJ counting red blood cell filled vessel structures. A significantly decreased capacity to form perfused vessels was found in implants containing shHIF-1α transfected MSPCs compared to untreated co-transplants. (n = 3, 5 high power fields 200x, mean ± SEM; ***p<0.0001).

### Oxygen-dependent Cell Culture, Clonogenicity and Re-oxygenation

ECFCs and MSPCs were grown and manipulated in an XVIVO system hypoxy workstation incubator (BioSpherix, New York, NY) set to 1% or 5% O_2_ in 95% humidified atmosphere containing 5% CO_2_ at 37°C. Media were equilibrated for 24 h to the required oxygen level in the hypoxy workstation before use. Conventional control cultures were done in ambient air (20% O_2_) in a standard incubator (Heraeus; Thermo Scientific) with 95% humidified atmosphere and 5% CO_2_ at 37°C. Oxygen content of media and cell supernatants was verified by blood gas analysis (Cobas B21, Roche, Burgess Hill, West Sussex, UK).

**Figure 5:**
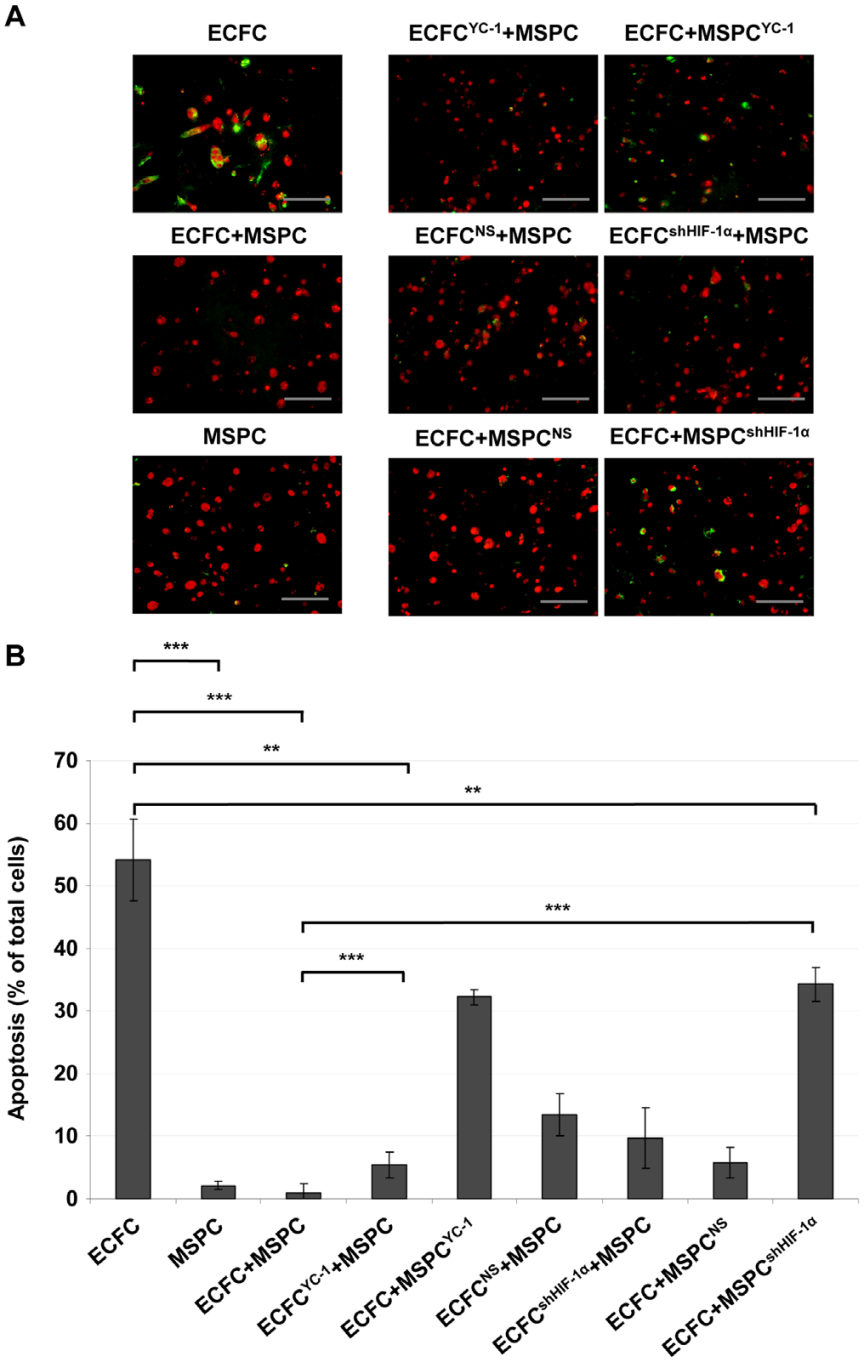
HIF-competent MSPCs are required to avoid premature ECFC apoptosis *in vivo*. (**A**) Apoptosis assay using TdT-mediated dUTP nick end labeling (TUNEL) of ECFC-only or MSPC-only transplants and treated or untreated ECFC+MSPC co-transplants, as indicated above each photograph. All plugs were explanted 24 h after implantation *in vivo*. DNA strand breaks of apoptotic cells were detected by a TUNEL assay kit (Promega) and nuclei were counterstained with propidium iodide (PI, red). Green fluorescence is due to FITC-labeled nucleotide binding to DNA strand breaks of apoptotic cells. Representative pictures from one experiment are shown (scale bar 100 µm, from at least three different donors performed per transplantation type as specified in the legends to Figures 3 and 4). (**B**) Apoptotic cells depicted as percentage of total cells ± SD with groups corresponding to the representative pictures in (A); five high power fields 200x; ***p<0.0001, **p<0.01).

ECFCs and MSPCs cultured at 1% and 5% O_2_ for 7 days were harvested in the hypoxy workstation and seeded for proliferation studies in 6-well plates at a density of 100 cells/cm^2^ or 30 cells/cm^2^, respectively. Clonogenicity of ECFCs and MSPCs was measured after seeding at indicated densities of 1–10 cells/cm^2^ in 55cm^2^ plates and either cultured in the previous oxygen conditions or re-oxygenated to 20% O_2_. After two weeks, cell numbers were measured by hemocytometry and colonies were fixed with paraformaldehyde within the hypoxy workstation, stained with crystal violet (Merck, Darmstadt, Germany) and enumerated as described earlier with ImageJ software (National Institutes of Health, Bethesda, MD) [17], [24]. Low proliferative potential (LPP) and high proliferative potential (HPP) colonies were defined as colonies comprising 51 to 500 and more than 500 cells, respectively [7], [17].

**Figure 6:**
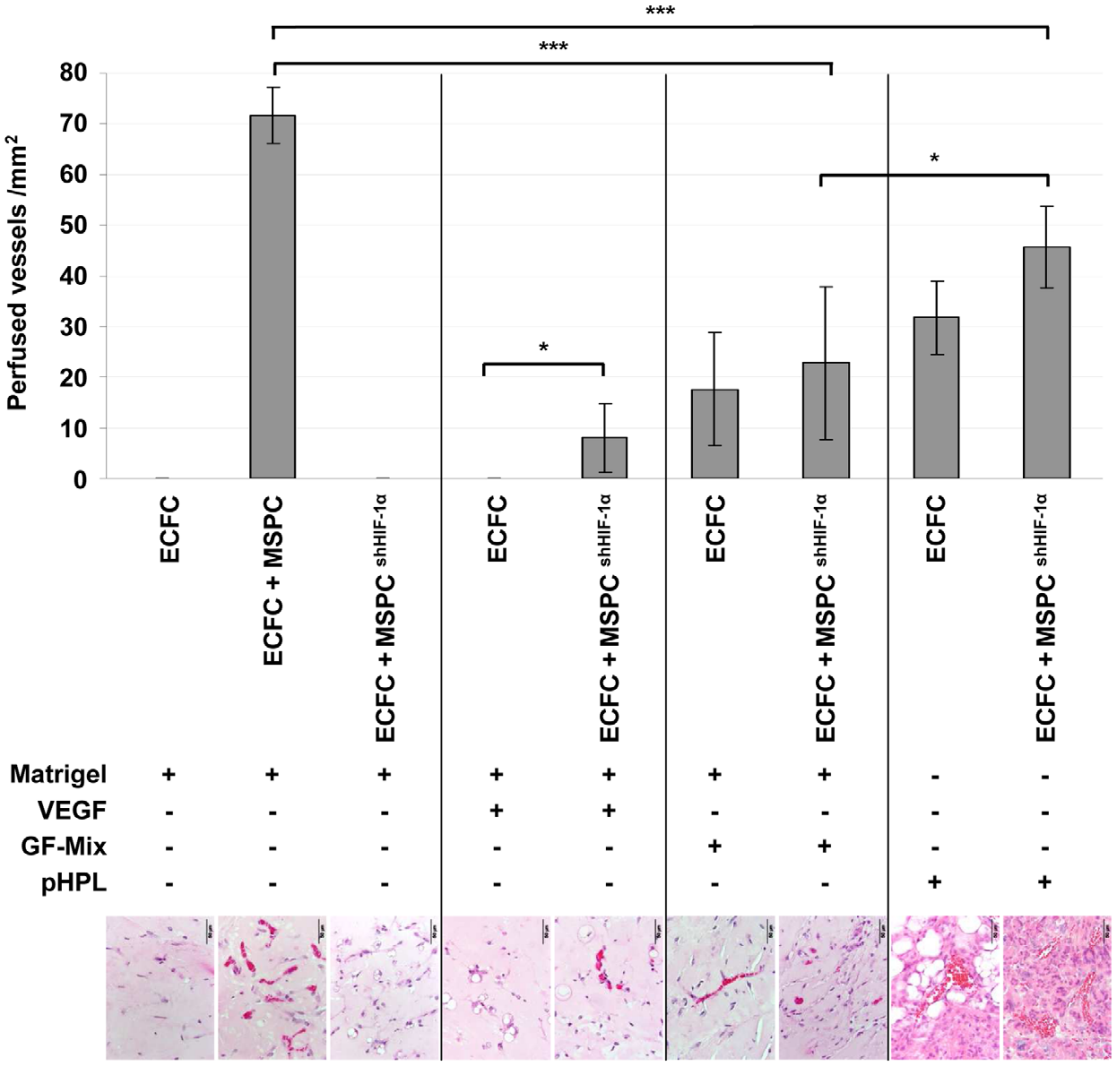
Angiogenic factors can partially substitute MSPC HIF-1α deficiency *in vivo*. ECFCs alone or in combination with MSPCs (either un-manipulated or after shHIF-1α knockdown) were (co)-transplanted in either Matrigel or pooled human platelet lysate (pHPL) gel with or without additional growth factors (GF) as specified. The GF-Mix comprised VEGF, EGF, IGF, FGF-2, hydrocortisone and ascorbic acid. Mean ± SD results of the number of perfused vessels counted in five high power fields (200x, at least two independent plugs and two independent animals). Microphotographs correspond to the treatment group (scale bar 50 µm; ***p<0.0001, *p<0.05).

### Angiogenesis Assay and Endothelial Wound Repair *in vitro*

To test the effects of different oxygen concentrations on vascular-like network formation, ECFCs were pre-cultured at 1%, 5% and 20% O_2_ for 7 days *in vitro*. After trypsinization, 180,000 ECFCs were re-suspended in 2 mL EGM-2/10% pHPL and seeded on 10 cm^2^ polymerized Matrigel/well (Angiogenesis assay kit; Millipore, Billerica, MA) according to the manufacturers’ instructions. Vascular-like networks (24 h) were documented with a Color View III camera on an Olympus IX51 microscope with the analySIS B acquisition software (all Olympus, Hamburg, Germany). Re-oxygenation to 20% O_2_ was done as described above. Tube number and length were determined using ImageJ software (http://rsbweb.nih.gov).

**Figure 7:**
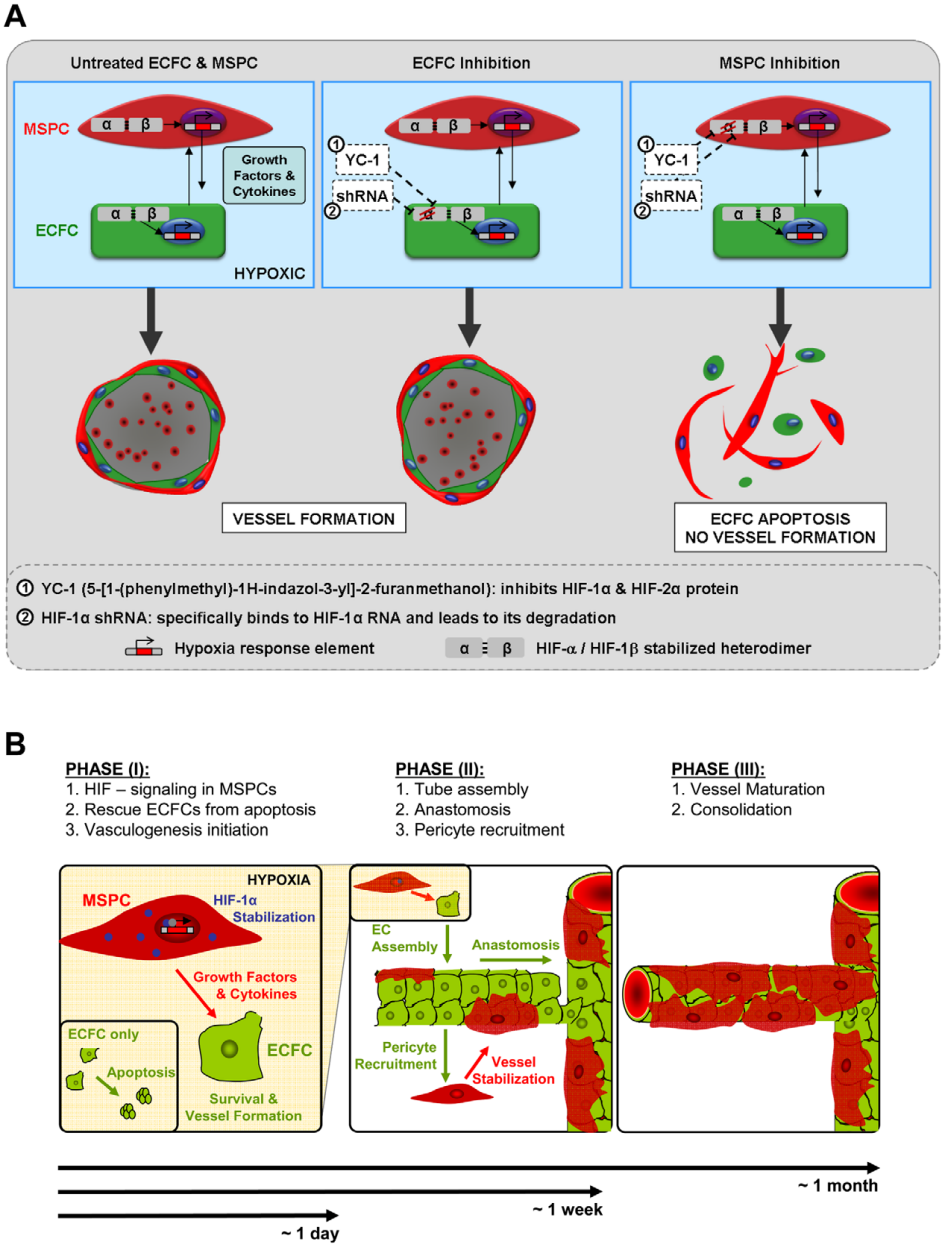
MSPCs are responsible for oxygen sensing and induction of neo-vasculogenesis. (**A**) The experimental strategy to delineate the hypoxia response used pre-treatment of either ECFCs or MSPCs before transplantation with YC-1 (inhibiting both HIF-1α as well as HIF-2α) or specific knockdown of HIF-1α RNA (by sh-RNA;). The surprising outcome of vessel formation despite the inhibition of ECFC hypoxia response and collapse of neo-vasculogenesis after MSPC inhibition is graphically illustrated. (**B**) Based on our current insight a model is proposed describing three temporarily distinct phases of adult vasculogenesis.

For endothelial wound repair studies, ECFCs were seeded at 1×10^5^ cells per 10 cm^2^ (Corning 6-well plate; Sigma) and cultured until confluence (approximately 4 d, 20% O_2_). After standardized scratch of the confluent cell layer with a 1,000 µL pipette tip (Gilson, Middleton, WI), cultures were introduced into the O_2_-controlled cell observer workspace (Carl Zeiss, Thornwood, NY) and the medium was replaced by pre-oxygenized fresh medium (1%, 5% or 20% O_2_) as indicated. Cell movement and proliferation were monitored by acquiring video sequences at 20 minute intervals covering a time period of up to 48 hours (Zeiss). Single cell proliferation was evaluated accurately by counting dividing cells on printouts of every video picture. A composite of three time-lapse videos covering 16 h of endothelial wound repair video sequences with cell divisions identified in red, green and yellow numbers as 1%, 5% and 20% O_2_, respectively (see Video S1). The area of wound repair was determined using ImageJ software.

### Experimental Neo-vasculogenesis *in vivo*

*In vivo* functionality was analyzed by resuspending 4×10^5^ bone marrow–derived MSPCs mixed with 1.6×10^6^ ECFCs per 0.3 mL in either ice-cold Matrigel (Millipore) or pHPL immediately before subcutaneous injection into the flank of immune-deficient NSG (NOD.Cg-Prdc^scid^ Il2rg^tm1Wjl^/SzJ) mice under general anesthesia as published [17]. Alternatively ECFCs and MSPCs were mixed with rat collagen and human fibronectin, allowed to coagulate, and were then implanted subcutaneously as a preformed plug in NSG mice as previously described [16]. Both ECFCs and MSPCs were derived from large-scale expansions providing consistent batches of cells after culture on up to 30,000 cm^2^ of culture area (12x 4-layered cell factories; Thermo Scientific) as previously published [17]. Mice were sacrificed by cervical dislocation at days 1, 7 and 14 after injection and plugs where explanted. To depict hypoxia, mice received intra-peritoneal injections of pimonidazole (60 mg/kg body weight; Hypoxyprobe, Burlington, MA) 30 minutes before plug explantation. Animal experiments were approved by the Animal Care and Use Committee as specified above.

### Immune Histochemistry and Immune Fluorescence

Sections (1.5 µm) of formalin-fixed (3.7% neutral buffered, overnight) paraffin-embedded plugs were de-paraffinized before antigen retrieval by heat (70°C/160 W, 40 min) at either high or low pH depending on the antibody followed by a descending alcohol series (2x xylol, 10 min; 1x ethanol 100%, 5 min; 1x ethanol 90%, 5 min; 1x ethanol 70%, 5 min; 1x ethanol 50%, 5 min; 1x PBS, 5 min). Endogenous peroxidases were blocked with H_2_O_2_ (10 min) and unspecific antibody binding with Ultra V Block (Thermo Scientific; 5 min) followed by mouse-on-mouse blocking (MOM, 1 h; Vector Laboratories, Burlingame, CA) and serum-free protein block (30 min; Dako, Glostrup, Denmark). Slides were incubated (30 min, RT) with unconjugated monoclonal mouse anti-human antibodies against Vimentin (clone: V9, 0.78 µg/mL, Dako), HIF-1α (clone: 54/HIF-1α, 10 µg/mL; BD Biosciences, San Jose, CA), CD31 (clone: JC70A, 5.15 µg/mL, Dako) or the appropriate amount of IgG1 (BD) control and developed with ultravision LP large-volume detection system horseradish peroxidase (HRP) polymer (Thermo Scientific) and diaminobenzidine (DAB) or alkaline phosphatase detection system using fast blue or fast red (Vector) according to manufacturer’s instructions. Avidin-biotin blocking (Vector) was used before staining with biotinylated monoclonal rat anti-mouse CD45 (clone: 30-F11, 5 µg/mL; R&D Systems, Minneapolis, MN) and biotinylated monoclonal rat anti-mouse IgG1 (BD) control and detected by streptavidin-HRP conjugate (Dako) and DAB. Pimonidazole was stained with 0.7 µg/mL anti-pimonidazole-FITC (Hypoxyprobe). For permanent staining a secondary HRP-conjugated anti-FITC antibody was applied and developed with DAB. Slides were counterstained with hematoxylin for ≤30 seconds.

For immune fluorescence slides were washed after antigen retrieval (5 min, PBS+0.01% v/v Triton X-100; Sigma), protein blocked (30 min; Dako) and stained overnight at 4°C with either biotinylated rat anti-mouse Ter119 (clone TER-119, 5 µg/mL, BD), biotinylated rat IgG2b isotype control (5 µg/mL, BD), HIF-1α (10 µg/mL, BD), or rabbit anti-human CD31 (clone: EPR3094, 1∶1,000; Abcam, Cambridge, UK), rabbit isotype control (5 µg/mL; Cell Signaling Technology, Danvers, MA), mouse anti-human alpha smooth muscle actin (clone: 1A4, 4.4 µg/mL, Dako), mouse IgG2a isotype control (4.4 µg/mL, Dako) all diluted in stain buffer, washed 3x (PBS+0.01% Triton X-100) and developed (120 min) with anti-mouse IgG-Alexa-546 (8 µg/mL, diluted in stain buffer; Invitrogen, Carlsbad, CA) or anti-rabbit IgG-Cy5 (2 µg/mL, Abcam) or streptavidin-conjugated alkaline phosphatase. After embedding in DAPI mounting media (4°C, minimum 60 min; Vector) pictures were taken with a Zeiss LSM510 Meta at 405, 543 and 633 nm excitation wavelengths. Collapsed stacks were reconstructed from z-stacks with Imaris software (Bitplane, Zurich, Switzerland).

To visualize HIF-1α protein in cell cultures at the single cell level, ECFCs and MSPCs were pre-cultured at 20% O_2_ to 75% confluence in 8-well glass chamber slides (Thermo Scientific). Medium was replaced by pre-oxygenized 1% or 5% medium in the hypoxy workstation or kept as 20% O_2_ and cells were cultured for 5 min – 24 h as specified. Incubation was stopped with ice cold PBS and fixed with ice cold paraformaldehyde (4%; 15 min, on ice) while still inside the hypoxy workstation. Immune cytochemistry was performed after H_2_O_2_ block using mouse anti-human HIF-1α antibody (BD) or mouse IgG1 control (10 µg/mL; BD) and visualized with HRP detection system (Thermo Scientific) with DAB according to manufacturers’ instructions. Cells were counterstained (15 sec) with hematoxylin and documented with a phase contrast microscope (Olympus).

### Vascular Endothelial Growth Factor (VEGF) Measurement in MSPC Supernatants

MSPCs were cultured in α-MEM containing 10% pHPL for 3 days at 1%, 5% or 20% O_2_. Supernatants were aspirated and sterile filtered (0.22 µm Steriflip-filter, Thermo Scientific). Supernatant was stored in aliquots at −80°C until measurement. VEGF concentration was determined using Bio-Plex Pro Assay (Bio-Rad, Laboratories, Hercules, CA) and analysed using the Bio-Plex-200 system (BioRad).

### Western Blot Analysis

ECFCs and MSPCs after 1%, 5% or 20% O_2_ culture for 6 h were washed once with ice cold PBS/Proteinase-inhibitor (Sigma) and then scraped in ice cold RIPA buffer (Bio-Rad) supplemented with proteinase and phosphatase-inhibitor (Thermo Scientific) and lysed under constant agitation on ice for 30 min. After sonication (15 sec, on ice; Hielscher, Teltow, Germany) and centrifugation, the protein-containing supernatants were aliquoted and frozen at −80°C. Protein content was determined with a Bradford assay (Bio-Rad) and OD was measured with a Spectramax (Molecular Devices, Sunnyvale, CA). Proteins (20 µg) were loaded and resolved in a 7.5% SDS-PAGE (Bio-Rad) in parallel to full-range rainbow™ molecular weight marker (GE Healthcare, Munich, Germany) and transferred onto a nitrocellulose membrane (Bio-Rad). HIF proteins were detected using HIF-1α (clone: 54/HIF1α, 0.5 µg/mL; BD), HIF-1β (clone: 29/HIF1β, 0.125 µg/mL; BD), HIF-2α (clone: A-5, 0.4 µg/mL; Santa Cruz, Santa Cruz, CA) antibodies compared to house-keeping protein control β-actin (clone: C4, 0.2 µg/mL; Santa Cruz) antibodies followed by anti-mouse-HRP antibody (Cell Signaling) and visualized with ‘super signal west pico luminol/enhancer developing solution’ (Thermo Scientific). Films were exposed to blots in a dark room (10 sec - 5 min). Developed films and blots were scanned overlayed to document the precise location of rainbow molecular weight marker bands (Figure S1E and S2).

### Hypoxia Response Modification *in vitro* and *in vivo*

The optimal dose of YC-1 [3-(5′hydroxymethyl-2′furyl)-1-benzylindazole] (100 µM, 5 min, 37°C; A.G. Scientific, San Diego, CA) blocking HIF-1α and HIF-2α proteins was titrated. Pre-treated MSPCs and ECFCs were washed 3x with PBS and combined with corresponding untreated partner cells for co-transplantation to study the impact of HIF inhibition in either cell type on vasculogenesis *in vivo*.

Lentiviral infections for HIF-1α depletion were carried out according to standard procedures for gene silencing. Briefly, 293T cells were co-transfected with pMD2.G and psPAX2 (Addgene, Cambridge, MA) along with either pGIPZ-HIF1α-shRNA or pGIPZ-scramble-shRNA lentiviral constructs (OpenBiosystems, Huntsville, AL) using JetPrime transfection reagent (Polyplus-transfection Inc., New York, NY) according to the manufacturer’s protocol. The transfection medium was replaced after 12 h with fresh DMEM/10% FBS (Sigma). After 48 h viral supernatants were collected and concentrated using Centricon Plus-70 filter units (Millipore). Bone marrow-derived MSPCs and peripheral blood-derived ECFCs were each infected in the presence of 8 µg/mL polybrene (Sigma). Two days after infection, stably transduced cells were selected with 2 µg/mL puromycin for 2 weeks resulting in a homogeneous population of virtually 100% TurboGFP-positive cells. To study the effect of different cytokines on vessel formation *in vivo*, Matrigel (Millipore) was mixed with 4 ng/mL of VEGF or a combination of VEGF, epidermal growth factor (EGF), insulin-like growth factor (IGF), basic fibroblast growth factor (FGF-2), hydrocortisone and ascorbic acid (all from Lonza EBM-2 Single Quots). Matrigel was replaced by pHPL before co-transplantation of either un-manipulated or HIF-1α knock down ECFCs and MSPCs wherever indicated. To block VEGF activity *in vivo*, mice were injected intraperitoneally every other day over a 7 or 14 day observation period starting at day zero immediately after co-transplantation of un-manipulated ECFCs and MSPCs as described above with Bevacizumab (Roche; 5 mg/kg per injection). Microvessel density was obtained from 5 high power fields (200x magnification) of HE stains from at least 2 independent donors and at least 2 independent plugs. Red blood cell-filled vessels were quantified by ImageJ software (http://rsbweb.nih.gov) and statistically evaluated by an unpaired student t-test. *p<0.05, **p<0.001, ***p<0.0001.

### Apoptosis Assay

Apoptosis of ECFCs and MSPCs in implants *in vivo* was analyzed with TdT-mediated dUTP nick end labeling (TUNEL; Dead End™ Fluoretric System; Promega, Madison, WI) according to manufacturer’s instructions. Nuclei were counterstained with propidium iodide (PI). As a positive control, slides were incubated for 10 min with 1U DNase 1 (Thermo Scientific). As a negative control, incubation buffer was prepared without rTdT. Staining was documented with a confocal microscope (LSM510 Meta, Zeiss).

### Statistics

Values are presented as mean ± standard deviation (SD). Comparisons of tube length in *in vitro* angiogenesis assays and wound repair assays were made by Mann-Whitney U test. Comparisons of microvessel density in the *in vivo* neo-vasculogenesis model were made by unpaired student t-test. Statistical differences were considered significant when the P-value was less than 0.05, very significant when P-value was less than 0.001 and extremely significant when P-value was less than or equal 0.0001.

## Results

### Mesenchymal Stem/progenitor Cells Sense Low Oxygen More Sensitively than Endothelial Progenitors *in vitro* and *in vivo*

In this study, the subcutaneous co-transplantation of human ECFCs with MSPCs into immune-deficient NSG mice as established previously was used as a vasculogenesis model to create stable perfused human mesenchymal cell-covered vessels [17]. The phenotype of applied cells was shown to be typical for MSPCs and ECFCs (Figure S3). Both ECFCs and MSPCs showed no reactivity to the hematopoietic marker CD45. Interestingly, one day after progenitor transplantation in advance of any vessel assembly and perfusion, plugs containing only ECFCs lack HIF-1α reactivity while implants containing MSPCs or the mixture of both progenitor cell types showed a strong nuclear HIF-1α signal (Figure 1A). When analyzing the human vessels in vascularized plugs after one week we observed that nuclear HIF-1α signals *in situ* were virtually restricted to mural cells (Figure S1A, B). To study the response to low oxygen more precisely at the single cell level over time, human ECFCs and MSPCs of different origin were exposed to reduced oxygen (5% O_2_ resembling the human venous oxygen level) and more severe hypoxia (1% O_2_) and compared to ambient air standard cell culture conditions (20% O_2_). Hypoxia was visualized by pimonidazole binding as described [31]. ECFCs from all tested sources showed cellular pimonidazole binding indicating hypoxia only at 1% O_2_. Surprisingly MSPCs and fibroblasts reproducibly bound pimonidazole already at 5% in addition to 1% O_2_ (Figure S1C). Nuclear HIF-1α resembling the pimonidazole results was found in the mesenchymal cells at 5% and 1% but not at 20% O_2_ and in ECFCs only at 1% O_2_ (Figure 1B). Kinetic analysis revealed nuclear HIF-1α protein stabilization in MSPCs after 1h at reduced O_2_. ECFCs accumulated HIF-1α starting after 2h and more prominently after 6h at 1% O_2_ (Figure S1D). Western blotting of cell lysates confirmed single cell results showing markedly increased HIF-1α stabilization in MSPCs when directly compared to ECFCs at 1% O_2_. We confirmed a stable HIF-1β expression in ECFCs and MSPCs and up-regulation of HIF-2α in ECFCs under more severe hypoxic conditions at 1% O_2_ (Figure 1C; see also Figure S1E).

### ECFCs Maintain a Quiescent State under Hypoxic Conditions *in vitro*

It is generally accepted that hypoxia maintains stemness [32]. It is not known whether the hypoxic environment during therapeutic vasculogenesis affects progenitor clonogenicity or function. Testing ECFC clonogenicity at 20% O_2_ standard conditions confirmed a complete hierarchy of LPP-ECFCs and HPP-ECFCs as previously reported [7], [17]. The colony number of ECFCs and MSPCs was stable under all O_2_ conditions tested but colony size progressively dropped with decreasing oxygen tension (Figure S4A, B). Progenitors exposed to 1% or 5% O_2_ experienced a hierarchy shift towards small colonies but resumed their clonogenic potential after re-oxygenation suggesting that they can maintain a quiescent state in a hypoxic environment and arguing against simple hypoxia-mediated damage (Figure S4C, D). In bulk cultures for large-scale expansion, O_2_ reduction diminished proliferation of both progenitor types progressively over time (Figure S4E-H). To determine the functionality of ECFCs under hypoxia, two standard assays were employed testing angiogenesis and endothelial wound repair *in vitro*. In a Matrigel assay, ECFCs pre-cultured at 5% and 20% O_2_ formed complex vascular networks. ECFCs pre-conditioned and tested at 1% O_2_ showed significantly reduced number and length of vessel-like structures (Figure 2A, B). When pre-cultured at 1% and subsequently tested at 20% O_2_ (re-oxygenation), ECFCs resumed their ability to form complex networks (Figure 2A). In addition, ECFCs almost closed the scratch area in an endothelial wound repair assay at 20% and 5% O_2_ within 24 hours. At 1% O_2_, the scratch area covered by ECFCs was significantly diminished and was accompanied by reduced proliferation (Figure 2C, D; Video S1).

### Neo-vasculogenesis *in vivo* Depends on MSPC HIF-1α Response and is Independent of ECFC Hypoxia Sensing

Neo-vasculogenesis was tested using a progenitor cell co-transplantation model in NSG mice as previously described [17]. Efficiency of experimental vasculogenesis *in vivo* in a previously established ratio of 80% ECFCs admixed with 20% MSPCs [17] was virtually independent of the carrier matrix protein (Figure S5, S6D). Interestingly, ECFCs alone largely failed to build complex vessel networks even after 14 days (Figure S6A, B). In the absence of human MSPCs the ECFCs progressively disappeared and were replaced by infiltrating mouse hematopoietic cells over time (Figure S6A- C). For reasons of comparability to existing studies [14], [15], [17], [33]–[36,36] Matrigel was chosen for further experiments in this study except when testing for the effect of human platelet-derived growth factors and cytokines present in pHPL.

In a hypoxic environment, cells respond in a cell-type specific manner by stabilizing HIF1-α or HIF2-α [37], [38]. To test which cell type is responsible for the hypoxia sensing during progenitor-derived neo-vasculogenesis *in vivo*, ECFCs and MSPCs were pre-treated with the small molecule hypoxia response inhibitor YC-1 [39] which blocks both HIF-1α and HIF-2α. Surprisingly, MSPC pre-treatment with YC-1 prior to co-transplantation with untreated ECFCs disabled vessel formation, whereas HIF-1α/HIF-2α inhibition in ECFCs did not significantly affect perfused vessel creation (Figure 3A, B). These results were confirmed by specifically silencing HIF-1α with small hairpin (sh)-RNA in either cell type before co-transplantation (Figure 4A). HIF-1α knock down in MSPCs abolished vessel formation significantly compared to transplants containing mock-transfected MSPCs. In accordance with YC-1 results, genetic HIF-1α ablation in ECFCs by sh-RNA did not result in the inhibition of vessel formation (Figure 4B, C).

### HIF-competent MSPCs Rescue ECFCs from Apoptosis

Based on our observation that HIF stabilization in response to hypoxia is an early event in MSPCs that is lacking in ECFCs, we asked whether apoptosis also plays a role early in the time course after cell transplantation. We found that the majority of ECFCs had already undergone apoptosis 24h post transplantation. MSPCs underwent virtually no apoptosis under hypoxic conditions *in vitro* or *in vivo* (Figure 5; Figure S7). Co-transplantation with HIF-competent MSPCs rescued ECFCs from apoptosis. Both HIF-1α knockdown by shRNA and YC-1-mediated HIF-1α/HIF-2α depletion in MSPCs led to a significant reduction of their anti-apoptotic effect. HIF depletion in ECFCs by either method did not influence early ECFC survival when co-transplanted with HIF-competent MSPCs (Figure 5).

HIF-1α stabilization in response to hypoxia leads to the transcription of hundreds of target genes including VEGF [37], [40]. Measuring MSPC cytokine secretion under different O_2_ conditions revealed a significant up-regulation of VEGF at hypoxic O_2_ levels. (Figure S8A). Blocking VEGF *in vivo* by repetitive intra peritoneal injection of bevacicumab largely ablated neo-vasculogenesis after progenitor co-transplantation (Figure S8B). To test whether VEGF represents the single dominant MSPC-derived pro-vasculogenic and anti-apoptotic factor in this system, ECFCs alone were transplanted subcutaneously in a matrix supplemented with a saturating concentration of VEGF. Whereas VEGF supplementation did not allow ECFCs to form vessels in the absence of HIF-competent MSPCs, it created a small but significant increase in perfused vessels two weeks after co-transplantation of ECFCs with HIF-1α-depleted MSPCs that otherwise reproducibly lacked the potential to contribute to vasculogenesis (Figure 6). To test if a multi-factorial system can rescue ECFCs from apoptosis and initiate vasculogenesis, we supplemented the Matrigel plugs with a defined mixture of vasculogenic growth factors established to support ECFC proliferation and function *in vitro* (VEGF, EGF, IGF, bFGF, hydrocortisone and ascorbic acid) [17]. This growth factor (GF)-mix enabled ECFCs alone as well as co-transplants of ECFCs with HIF-1α-depleted MSPCs to form perfused vessels (Figure 6). Vasculogenesis in the presence of the GF-mix was still significantly less efficient than that initiated in the presence of HIF-competent MSPCs.

Sole ECFCs injected in a growth factor-rich pHPL led to the formation of enlarged and lagoon-like vessels which is largely reminiscent of a pathology found in several vascular anomalies including hemangioma (Figure S5). Based on this observation we also tested co-transplants of ECFCs with HIF-1α-depleted MSPCs in pHPL. This resulted in the highest number of perfused vessels formed by ECFCs co-transplanted with HIF-depleted MSPCs, but this combination was still significantly less efficient than co-transplantation of ECFCs with HIF-competent MSPCs (Figure 6).

## Discussion

Understanding the cross-talk of endothelial and mural cells in response to the hypoxic environment and deciphering the underlying mechanisms of new vessel formation is of great relevance for the development of novel strategies aimed at therapeutic vasculogenesis. Despite promising experimental data, approaches focusing on endothelial progenitors have been of limited efficiency in clinical trials, both for therapeutic vasculogenesis and for anti-angiogenic therapy [10]. It has only recently been recognized that endothelial lineage cells require interaction with MSPCs to efficiently build perfused vessels *in vivo* [11], [12], [14], [17], [18], [33].

Here we report that perivascular MSPCs sense hypoxia earlier and more efficiently than ECFCs, which build the inner vessel wall. HIF-1α stabilization and transition to the nucleus is a key event initiating gene expression in response to hypoxia [21]. One explanation for the lack of endothelial HIF-1α accumulation in our transplantation model would be its immediate degradation during rising oxygen levels following the initiation of perfusion. Our observation that ECFCs lack detectable HIF-1α as soon as one day after transplantation prior to vessel assembly and plug perfusion, argues against such a simplified view. Two independent strategies were therefore utilized to delineate the role of HIF as a key regulatory element during vasculogenesis (Figure 7A). Unexpectedly, neither HIF-1α knockdown by shRNA nor pharmacologic HIF-1α and HIF-2α deletion by YC-1 in ECFCs affected patent vessel formation. In contrast, HIF-1α knockdown or YC-1-mediated inhibition in MSPCs completely abrogated experimental vasculogenesis. These results clearly demonstrate that HIF-1α stabilization in MSPCs but not in ECFCs is a crucial event in therapeutic vasculogenesis. It will be of great interest to study the impact of this endothelial-mesenchymal crosstalk on the metabolic balance of the two cell types under hypoxic conditions. This may not be limited solely to vascular regeneration, as cardiac regeneration was also found to depend on the functionality of endothelial and smooth muscle precursors in a model of bone marrow-derived cell therapy [41]. Depending on the source of MSPCs and the balance between ECFC and MSPC, developmental programs can be activated that allow the formation of human bone and marrow niche. This system has already enabled us to construct a genetically controlled hematopoietic microenvironment in which HIF-1α controls the engraftment of normal and malignant blood cells [42].

Another aspect of our studies is the role of HIF-2α as an alternative O_2_-responsive transcription factor in endothelial lineage cells [43], [44]. Our observation that YC-1 pre-treatment of ECFCs does not affect vessel formation in this system does not address the specific function of HIF-2α during vessel normalization [44]. Endothelial-specific *Hif-2*α deletion in mice resulted in a virtually normal vascular phenotype except for abnormal vessel permeability under steady state conditions, while increased vessel formation was observed during vascular regeneration and unstable tumor vascularization [45], [46]. Although we did not analyze neo-vessel stability and/or permeability in this study, the sparse pericyte distribution around some of the vessels derived from YC-1 pre-treated ECFCs (Figure 3A) may be reminiscent of the HIF-2α deficient phenotype. Lack of up-regulation of HIF-2α in MSPCs during HIF-1α knockdown argues against a critical role of HIF-2α in MSPC function.

The diminished expansion of endothelial and mesenchymal progenitor cells as observed in our study under stringently reduced oxygen is of relevance for cell propagation for regenerative purposes. Hypoxic pre-conditioning strategies, despite maintaining clonogenicity, would limit net cell expansion. The phenomenon that ECFCs, after silencing in hypoxia, resume their ability to form complex vascular networks after re-exposure to 20% O_2_ further substantiated the argument that ECFCs can maintain a functionally quiescent state in a hypoxic environment capable of being resumed when re-oxygenated. The reduced proliferation and diminished endothelial wound repair as observed under hypoxic conditions *in vitro* support the notion that insufficient ECFC hypoxia response can translate into a reversible functional deficit. Based on our data we speculate that MSPCs or perhaps other HIF-competent stromal cells can function to rescue vascular regeneration on demand. It has been previously demonstrated that adipose tissue-derived stem cells can function as pericytes when co-transplanted with cord blood-derived endothelial progenitors. The mural cells prevent vessel regression around day 14 post implantation by a platelet-derived growth factor (PDGF)-independent mechanism that reduced apoptotic endothelial cell death thus leading to robust vessel assembly [18]. In our *in vivo* model, ECFC apoptosis in the hypoxic environment was prevented by co-transplantation of HIF-competent but not of HIF-deficient MSPCs. Recently, we also found that the anti-apoptotic activity of MSPCs can rescue human-induced pluripotent stem (iPS) cells from apoptosis resulting in their long-term engraftment in a preclinical pig model of myocardial infarction [47].

We conclude that ECFC/MSPC co-transplantation represents a valuable alternative to current endothelial cell therapy strategies for vessel repair and tissue engineering. The ideal carrier for cell application still needs to be determined. A humanized matrix based on pHPL was applied in comparison to a collagen/fibronectin mixture and Matrigel representing an experimental standard. The fact that progenitor cells admixed with pHPL solution can be injected with a regular syringe may offer a certain technical advantage compared to collagen/fibronectin, which requires solidification before surgical implantation of preformed plugs. Injectable type I collagen, fibrin and puramatrix have also been shown to support early vessel formation after two-cell-type implantation [48]. Another recent study showed that co-transplantation of ECFCs with MSPCs in a mouse model was most efficient in regenerating perfusion after hindlimb ischemia [49]. Application of either cell type alone was less efficient but revealed distinct mechanisms by which ECFCs and MSPCs contribute to vascular and tissue regeneration [49]. Whether physical contact of ECFCs and MSPCs is necessary to exert the supportive effects of MSPCs remains to be determined. In the presence of HIF-silenced MSPCs *in vivo*, ECFCs were not capable of performing their vasculogenic capacity. In this context it is interesting to note that part of the effect of HIF-competent MSPCs could be mimicked by pHPL (Figure 6). This supports the hypothesis that the effect of MSPCs is mediated to a reasonable extend by HIF-mediated protein secretion. Whether platelet-derived growth factors in platelet-rich plasma or pHPL could be utilized as an adjuvant improving or even substituting cell therapy remains to be determined [50], [51].

The fact that continuous VEGF blockade virtually ablated progenitor-derived neo-vasculogenesis was not unexpected. Because VEGF depletion *in vivo*, despite negatively regulating mural cell function, lacks sustained clinical efficiency [10], [34], [44], its combination with MSPC-targeted therapies offers an alternative strategy.

Our unexpected observation that therapeutic vasculogenesis can occur independent of endothelial HIF function *in vivo* further strengthens the need to re-examine current concepts in vascular regeneration using innovative tracking strategies at the single cell level [52]. Admitting that EC-targeted deletion of HIF-1α or HIF-2α as published previously [45], [53] resulted in a surprisingly unremarkable phenotype, except for its profound disturbance of tumor angiogenesis, may imply that tumor vessel growth as compared to regenerative vasculogenesis (in this study) and angiogenesis [36] follow different developmental programs with a certainly underestimated impact of the perivascular compartment. However, also in the *Hif-1*α knockout animals, the severe vascular defects resulting in embryonic lethality were spatially correlated with perivascular mesenchymal cell death and not associated with VEGF deficiency [54]. It remains to be determined whether MSPCs already express HIF-1α at 20% O_2_ as a cell culture artifact or whether there is a physiologically rational pathway activated in MSPCs fitting them with the competence to react to hypoxia earlier and/or more sensitively than other cells. Coagulation factors like tissue factor and thrombin are known to interfere with blood vessel development and homeostasis [55]. Angiotensin II, bacterial lipopolysaccharides (LPS) and various growth factors and cytokines are non-hypoxic stimuli that can regulate HIF expression [56]. Proteases also play a key role in the later phase of anastomosis of newly formed vessels during a process called ‘wrapping and tapping’ which realizes the connection with the existing vasculature [57]. The existence of an indirect mural cell-mediated oxygen sensing pathway early during the initiation of vasculogenesis as observed in our study prompted us to propose a model which incorporates these new aspects of vasculogenesis (Figure 7B). Further experiments are required to evaluate this model.

The recent discovery that fibroblast growth factor-9 can act on stromal precursors stabilizing vessel outgrowth highlights the role of mural cells during sprouting angiogenesis [36]. Hematopoietic and tumor cells can also act in concert with endothelial and mesenchymal progenitors to initiate, arrange and support vessel formation in hypoxic environments [3], [8], [10], [35], [41], [44]. Understanding the peculiar role stromal cells can assume in that orchestrated interplay will help to critically develop more efficient pro- and anti-angiogenic strategies.

## Supporting Information

Figure S1 **Nuclear HIF-1α signal** ***in vitro*** **and** ***in vivo*** **.** (**A**) Immune histochemical staining of matrigel plugs containing ECFCs/MSPCs 7 days (d) after co-transplantation. Plugs were explanted and sections were stained with anti-HIF-1α (brown; arrow heads), anti-human CD31 (red) and co-stained with hematoxylin (blue). (**B**) Immune fluorescence staining d7 after co-transplanting ECFCs/MSPCs in matrigel with anti-HIF-1α (white), anti-human alpha smooth muscle actin (SMA, red), and counterstained with DAPI (blue), anti-human CD31 (green). White arrow heads mark nuclear HIF-1α signals and arrows unspecific background fluorescence of mouse red blood cells. (**C**) Hypoxyprobe (pimonidazole, green; DAPI nuclear stain in blue) analysis of MSPCs from bone marrow (BM), umbilical cord (Cord), neonatal and adult fibroblasts directly compared to ECFCs from umbilical cord blood (UCB), cord, normal and cardiovascular disease patient-derived (CVD) peripheral blood (PB) cultured at indicated O_2_ levels as described in the methods section. Scale bar 100 µm. (**D**) ECFCs and MSPCs were cultured for indicated intervals at indicated O_2_. Fixed cells were stained with anti-HIF-1α. ImageJ (http://rsbweb.nih.gov) processing was used to obtain the transformed red signal. Original data are on file. ECFC start to stabilize HIF-1α in their nucleus after 2 h at 1% O_2_ but not at 5 or 20% O_2_. MSPCs stabilize HIF-1α after 1h at 1% and 5% O_2_. (**E**) Western blot analysis of ECFC and MSPC total cell lysates after 6h at indicated O_2_. Blots were incubated with HIF-1α, HIF-1β, HIF-2α or β-actin (β-Act) antibodies. Three representative blots scanned in overlay with exposed films. Scissors mark cuts to separately stain β-actin (β-Act) from the same blot. Areas shown in Fig. 1B are boxed.
(TIF)

Figure S2 **Specific knock-down of HIF-1α in MSPCs and ECFCs.** Total cell lysates of untreated control (**-**) ECFCs and MSPCs or after infection with either pGIPZ-HIF1alpha-shRNA (shHIF-1α) or non-specific pGIPZ-scramble-shRNA (NS) were separated by SDS-PAGE after 6 hours of incubation at 1% O_2_. Blots were stained with either HIF-1α, HIF-2α, HIF-1β or β-actin (β-Act). Three representative blots scanned in overlay with exposed films are shown. Scissors mark cuts to separately stain β-Act from the same blot. Areas shown in Figure 5A are boxed.
(TIF)

Figure S3 **Phenotypic characterization of MSPCs and ECFCs.** The phenotype of MSPCs and ECFCs was characterized by flow cytometry as described previously (n>5) [17]. MSPCs and ECFCs can be distinguished by their dissimilar expression of CD90, CD31 and CD34. Both ECFCs and MSPCs show no reactivity with the hematopoietic marker CD45.
(TIF)

Figure S4 **Progenitor clonogenicity and long-term proliferation: proliferative quiescence under reduced O_2_ and recapitulation after re-oxygenation.** (**A, B**) For colony assays 10 ECFCs/cm^2^ (n = 3) or 3 MSPCs/cm^2^ (n = 2) were seeded in 55 cm^2^ colony plates and grown for 14 days (d) at 1%, 5% or 20% O_2_. Colony number and cell number were documented. Culture plates show typical colonies at 1%, 5% and 20% O_2_ derived from the same ECFC or MSPC starting population, respectively, and stained with crystal violet as described in the methods section. (**C**) For re-oxygenation (+ reoxy) population doublings (PD) of ECFCs and MSPCs cultured under 1%, 5% and 20% O_2_ for 12 d were compared with PDs of ECFCs and MSPCs pre-cultured at 1 or 5% O_2_ for 7 d and then cultured at 20% O_2_ for another 12 d (mean ± SD; n = 3). Corresponding representative crystal violet-stained colony plates are shown positioned below their corresponding O_2_ conditions. (**D**) ECFC hierarchy was assessed after 14 d of culture at 1%, 5% and 20% O_2_ directly compared to ECFCs pre-cultured for 7 d at 1% or 5% O_2_ and subsequently for another 12 d at 20% O_2_ (+reoxy) by photo documenting all colonies per plate and semi-automatically counting every single cell per scanned colony as described previously using the ImageJ software (http://rsbweb.nih.gov). One representative experiment is shown. (**E**) To determine population doublings (PD) per passage 100 ECFCs/cm^2^ (n = 5) or 30 MSPCs/cm^2^ (n = 2) were seeded in 75 cm^2^ culture flasks and grown for 14 d at 1%, 5% or 20% O_2_. (**F, G)** Cumulative PDs were calculated after long term culture (3×14 d) at 1%, 5% or 20% O_2_ for (**F**) ECFCs or (**G**) MSPCs. (**H**) Representative ECFC and MSPC colonies are shown after 14 d incubation at 1%, 5% or 20% O_2_ after crystal violet stain (scale bar 5 mm).
(TIF)

Figure S5 **Patent vessel formation depends on functioning MSPCs and is virtually matrix- independent.** ECFCs (1.6×10^6^) together with MSPCs (4×10^5^) for co-transplantation or sole ECFCs (2×10^6^) were re-suspended in ice cold matrigel, collagen/fibronectin (Coll/Fn), or pooled human platelet lysate (pHPL), respectively. Aliquots of matrigel or pHPL were injected and preformed collagen/fibronectin plugs were implanted subcutaneously as described in methods in detail into the flank of NSG mice. Mice were sacrificed on day (d) 7 and 1.5 µm plug sections were either stained with hematoxylin and eosin (HE) or processed for anti-human vimentin immune histochemistry (h.Vimentin; hematoxylin counterstain, blue; see methods for details and references). Control plugs were also explanted at d 1 (see Figure S4D). The higher cell density despite equal cell input in pHPL implants results from more intense contraction of the matrix *in vivo* compared to matrigel.
(TIF)

Figure S6 **Patent vessel formation depends on MSPC presence and is virtually matrix-independent.** (**A, B**) ECFCs alone or MSPCs+ECFCs (ratio 20∶80) were re-suspended in matrigel and injected subcutaneously into immune deficient NSG (NOD.Cg-Prkdcscid Il2rgtm1Wjl/SzJ) mice. Plugs where explanted at days (d) 1, 7 and 14. (**A**) Hematoxylin and eosin. (**B**) Mesodermal origin was probed with anti-human vimentin (brown; nuclei blue, hematoxylin). Implants after ECFC+MSPC co-transplantation showed vimentin^+^ human vessel formations (d7 & d14) compared to implants of ECFC alone showing not more than rare small vessel-like structures and declining human cell number. (**C**) Infiltrating vimentin-negative (non-human) cells in ECFC plugs were mouse CD45^+^ (mouse hematopoietic) cells already 7 d after transplantation. (**D**) Hematoxylin/eosin staining to visualize cells in different extracellular matrices 1 d after transplantation. MSPCs + ECFCs (top row) or ECFCs only (bottom row) were re-suspended in matrigel or pooled human platelet lysate (pHPL) and injected subcutaneously (6.6×10^6^/mL; ratio 20∶80; injection volume 300 µL) into immune-deficient NSG mice (NOD.Cg-Prkdcscid Il2rgtm1Wjl/SzJ; n ≥3). Preformed collagen/fibronectin plugs containing equal cell compositions were implanted (n = 3).
(TIF)

Figure S7 **Anti-VEGF treatment inhibits vessel formation** ***in vivo*** **.** (**A**) VEGF concentration in supernatant of MSPCs cultured for 3 days at 1%, 5% and 20% O_2_ showed increasing VEGF levels with decreasing oxygen concentration (mean ± SD; n = 3). (**B**) Anti-VEGF treatment inhibited vessel formation in matrigel plugs. After subcutaneous co-transplantation of MSPCs and ECFCs (ratio 20∶80) into NSG (NOD.Cg-Prdc^scid^ Il2rg^tm1Wjl^/SzJ) mice recipients were injected i.p. with 5 mg/kg of the therapeutic anti-human VEGF antibody Bevacizumab every other day (d) starting d1. Hematoxylin/eosin staining showed limited cell arrangement but no human vessel formation after one week (d7; three doses of antibody). After seven doses of antibody (d14) some tiny vessels could be observed (arrows). Histology magnification is indicated by scale bar (200 µm). Macro-photography inserts show the freshly explanted pale plugs.
(TIF)

Figure S8 **Apoptosis of MSPCs under hypoxia** ***in vitro.*** Representative flow cytometry dot blot showing Annexin V binding to phosphatidylserine of apoptotic MSPCs combined with propidium iodide (PI) labeling after culture under hypoxic conditions (1% oxygen) for 8 days. Annexin V^+^/PI^-^(Annexin single positive) MSPCs represent apoptotic cells with an intact membrane excluding PI. Annexin V^+^/PI^+^ (double positive) MSPCs represent terminally dead cells which accumulate PI. (FSC, forward light scatter; SSC, side scatter).
(TIF)

Video S1 **Related to** **Figure 2:** **Wound healing assay** ***in vitro*** **.** Wounding an ECFC-derived monolayer in a scratch assay was used to monitor endothelial wound repair under hypoxia (1% O_2_) as compared to reduced (5% O_2_) and ambient air (20% O_2_) standard laboratory test conditions. Running numbers refer to cell divisions and sum up to the total number of single cell divisions for each oxygen concentration.
(7z) Click here for additional data file.
