# Supplementary figures and images for: Oxygen Sensing Mesenchymal Progenitors Promote Neo-Vasculogenesis in a Humanized Mouse Model In Vivo
Source: PLoS One. 2012 Sep 7;7(9):e44468. doi: 10.1371/journal.pone.0044468 (PMC3436890; doi:10.1371/journal.pone.0044468)

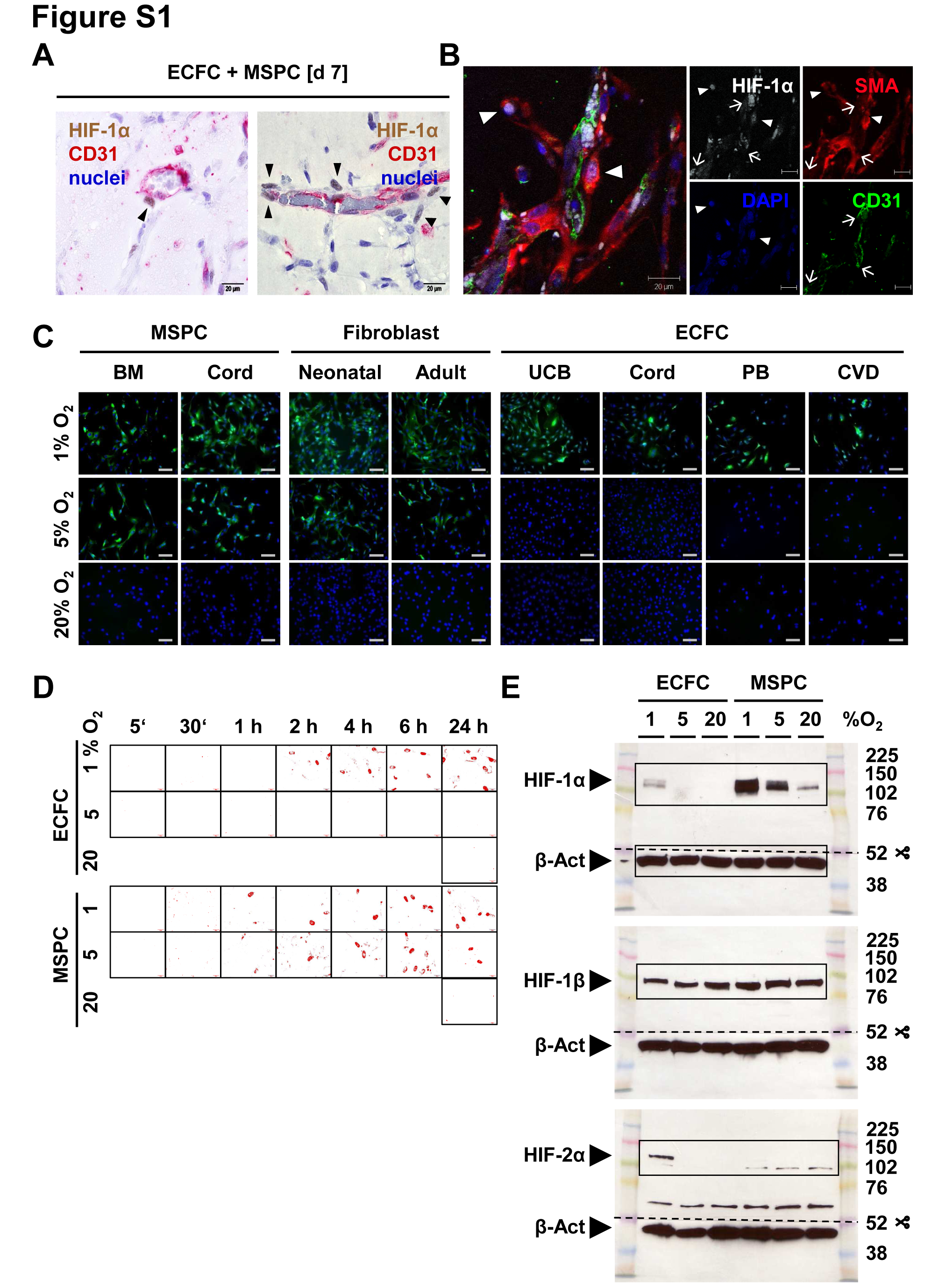

Supplement: Figure S1 — Nuclear HIF-1α signal in vitro and in vivo . (A) Immune histochemical staining of matrigel plugs containing ECFCs/MSPCs 7 days (d) after co-transplantation. Plugs were explanted and sections were stained with anti-HIF-1α (brown; arrow heads), anti-human CD31 (red) and co-stained with hematoxylin (blue). (B) Immune fluorescence staining d7 after co-transplanting ECFCs/MSPCs in matrigel with anti-HIF-1α (white), anti-human alpha smooth muscle actin (SMA, red), and counterstained with DAPI (blue), anti-human CD31 (green). White arrow heads mark nuclear HIF-1α signals and arrows unspecific background fluorescence of mouse red blood cells. (C) Hypoxyprobe (pimonidazole, green; DAPI nuclear stain in blue) analysis of MSPCs from bone marrow (BM), umbilical cord (Cord), neonatal and adult fibroblasts directly compared to ECFCs from umbilical cord blood (UCB), cord, normal and cardiovascular disease patient-derived (CVD) peripheral blood (PB) cultured at indicated O2 levels as described in the methods section. Scale bar 100 µm. (D) ECFCs and MSPCs were cultured for indicated intervals at indicated O2. Fixed cells were stained with anti-HIF-1α. ImageJ (http://rsbweb.nih.gov) processing was used to obtain the transformed red signal. Original data are on file. ECFC start to stabilize HIF-1α in their nucleus after 2 h at 1% O2 but not at 5 or 20% O2. MSPCs stabilize HIF-1α after 1h at 1% and 5% O2. (E) Western blot analysis of ECFC and MSPC total cell lysates after 6h at indicated O2. Blots were incubated with HIF-1α, HIF-1β, HIF-2α or β-actin (β-Act) antibodies. Three representative blots scanned in overlay with exposed films. Scissors mark cuts to separately stain β-actin (β-Act) from the same blot. Areas shown in Fig. 1B are boxed. (TIF) [file pone.0044468.s001.tif]

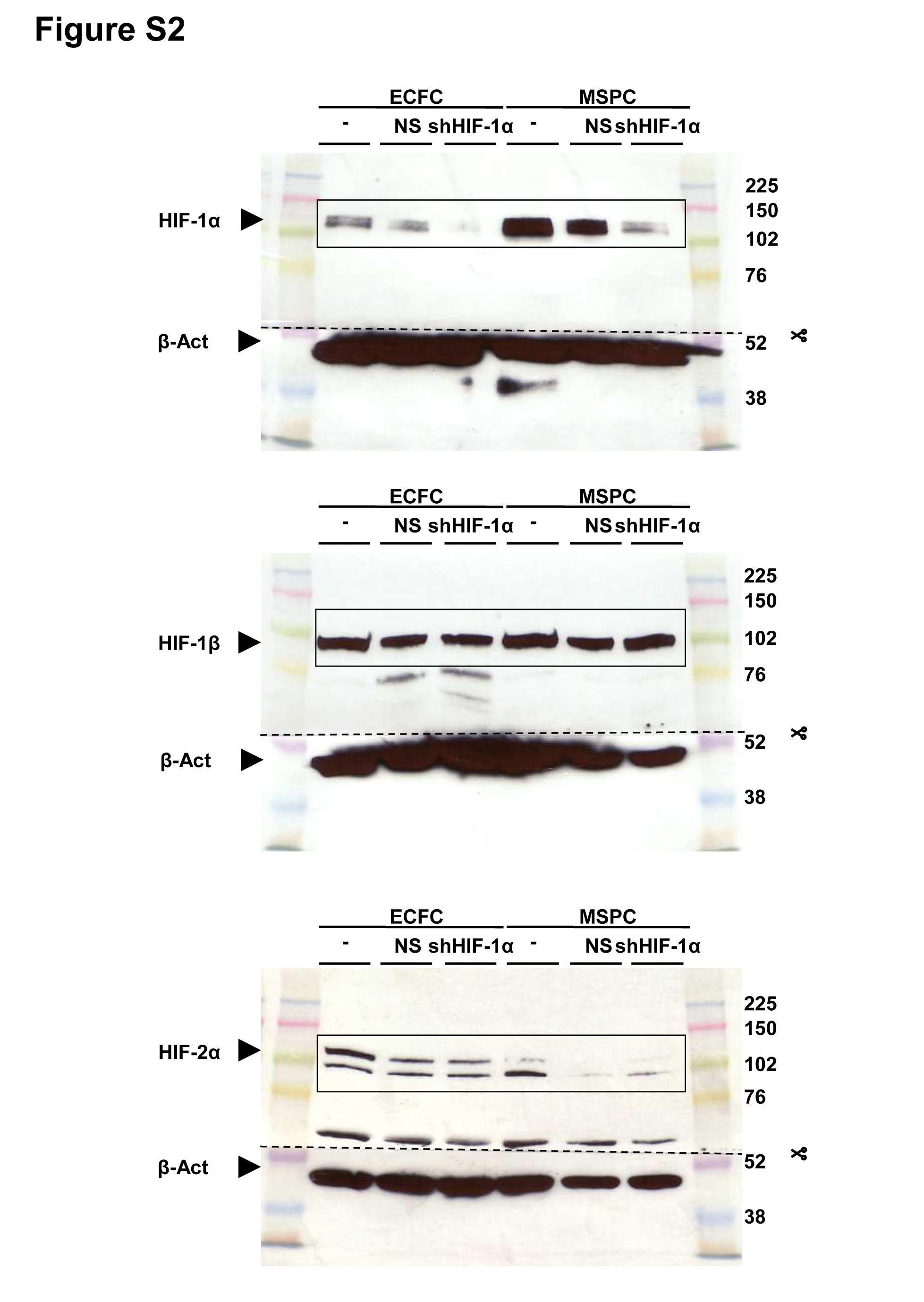

Supplement: Figure S2 — Specific knock-down of HIF-1α in MSPCs and ECFCs. Total cell lysates of untreated control (-) ECFCs and MSPCs or after infection with either pGIPZ-HIF1alpha-shRNA (shHIF-1α) or non-specific pGIPZ-scramble-shRNA (NS) were separated by SDS-PAGE after 6 hours of incubation at 1% O2. Blots were stained with either HIF-1α, HIF-2α, HIF-1β or β-actin (β-Act). Three representative blots scanned in overlay with exposed films are shown. Scissors mark cuts to separately stain β-Act from the same blot. Areas shown in Figure 5A are boxed. (TIF) [file pone.0044468.s002.tif]

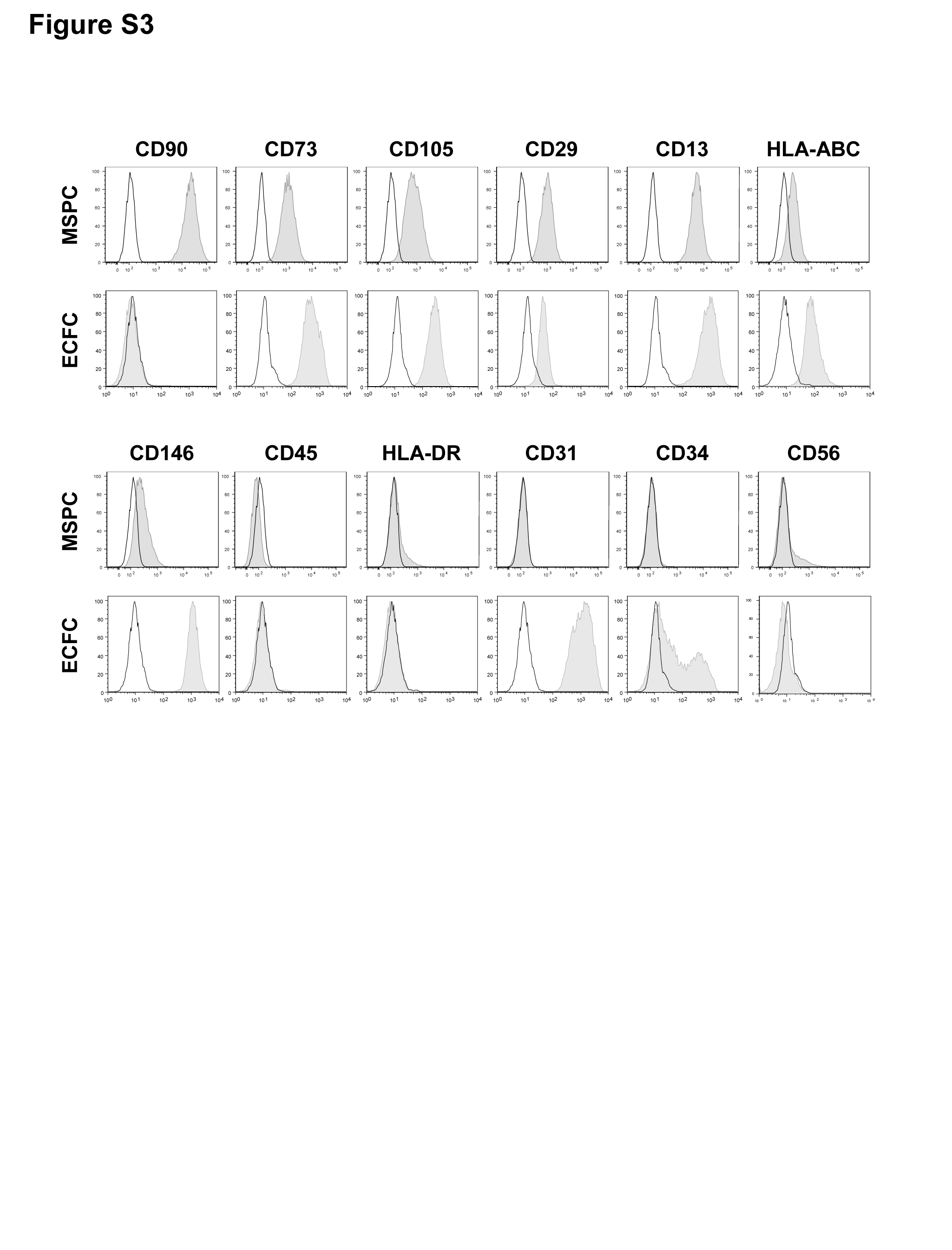

Supplement: Figure S3 — Phenotypic characterization of MSPCs and ECFCs. The phenotype of MSPCs and ECFCs was characterized by flow cytometry as described previously (n>5) [17]. MSPCs and ECFCs can be distinguished by their dissimilar expression of CD90, CD31 and CD34. Both ECFCs and MSPCs show no reactivity with the hematopoietic marker CD45. (TIF) [file pone.0044468.s003.tif]

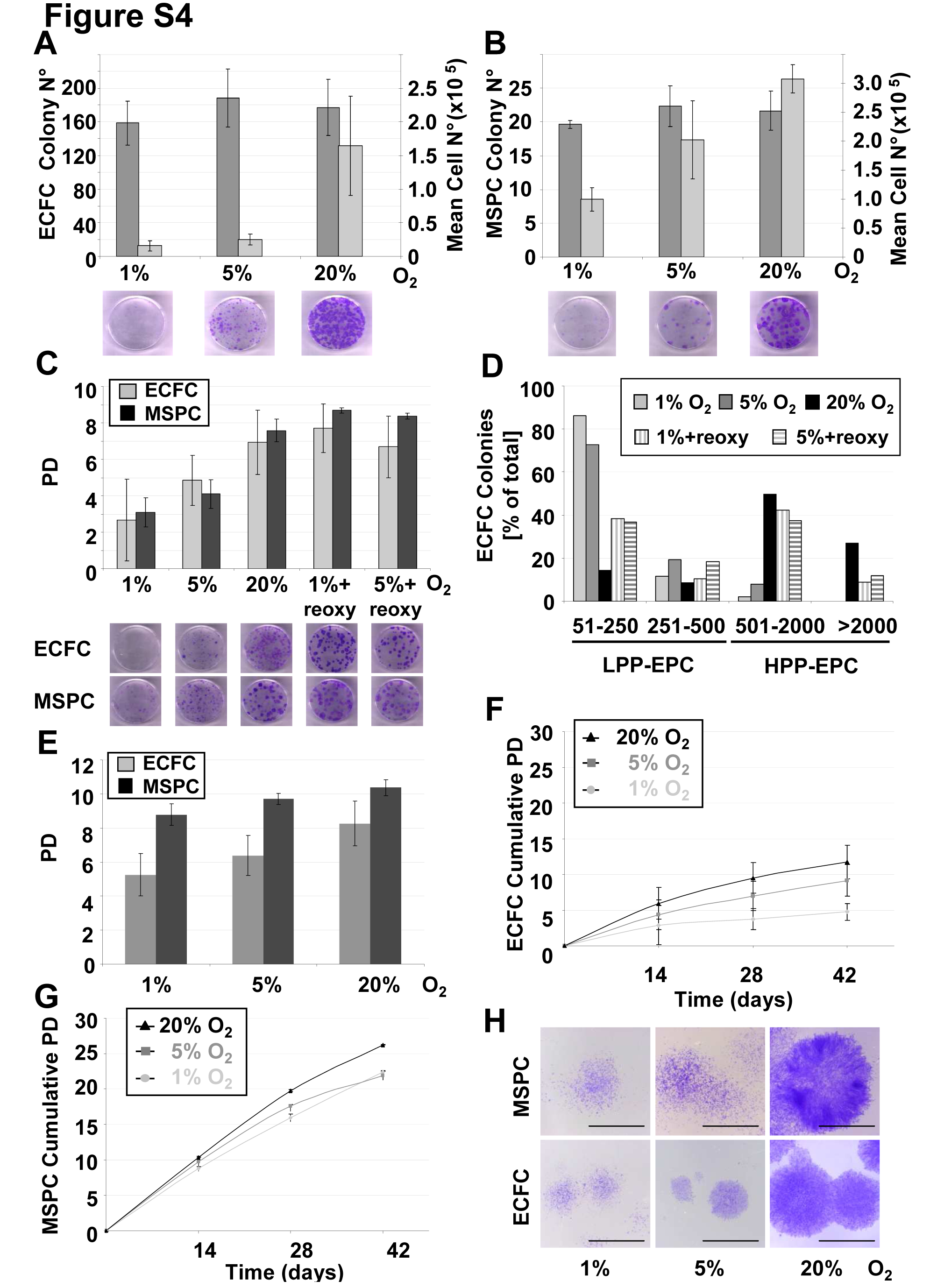

Supplement: Figure S4 — Progenitor clonogenicity and long-term proliferation: proliferative quiescence under reduced O2 and recapitulation after re-oxygenation. (A, B) For colony assays 10 ECFCs/cm2 (n = 3) or 3 MSPCs/cm2 (n = 2) were seeded in 55 cm2 colony plates and grown for 14 days (d) at 1%, 5% or 20% O2. Colony number and cell number were documented. Culture plates show typical colonies at 1%, 5% and 20% O2 derived from the same ECFC or MSPC starting population, respectively, and stained with crystal violet as described in the methods section. (C) For re-oxygenation (+ reoxy) population doublings (PD) of ECFCs and MSPCs cultured under 1%, 5% and 20% O2 for 12 d were compared with PDs of ECFCs and MSPCs pre-cultured at 1 or 5% O2 for 7 d and then cultured at 20% O2 for another 12 d (mean ± SD; n = 3). Corresponding representative crystal violet-stained colony plates are shown positioned below their corresponding O2 conditions. (D) ECFC hierarchy was assessed after 14 d of culture at 1%, 5% and 20% O2 directly compared to ECFCs pre-cultured for 7 d at 1% or 5% O2 and subsequently for another 12 d at 20% O2 (+reoxy) by photo documenting all colonies per plate and semi-automatically counting every single cell per scanned colony as described previously using the ImageJ software (http://rsbweb.nih.gov). One representative experiment is shown. (E) To determine population doublings (PD) per passage 100 ECFCs/cm2 (n = 5) or 30 MSPCs/cm2 (n = 2) were seeded in 75 cm2 culture flasks and grown for 14 d at 1%, 5% or 20% O2. (F, G) Cumulative PDs were calculated after long term culture (3×14 d) at 1%, 5% or 20% O2 for (F) ECFCs or (G) MSPCs. (H) Representative ECFC and MSPC colonies are shown after 14 d incubation at 1%, 5% or 20% O2 after crystal violet stain (scale bar 5 mm). (TIF) [file pone.0044468.s004.tif]

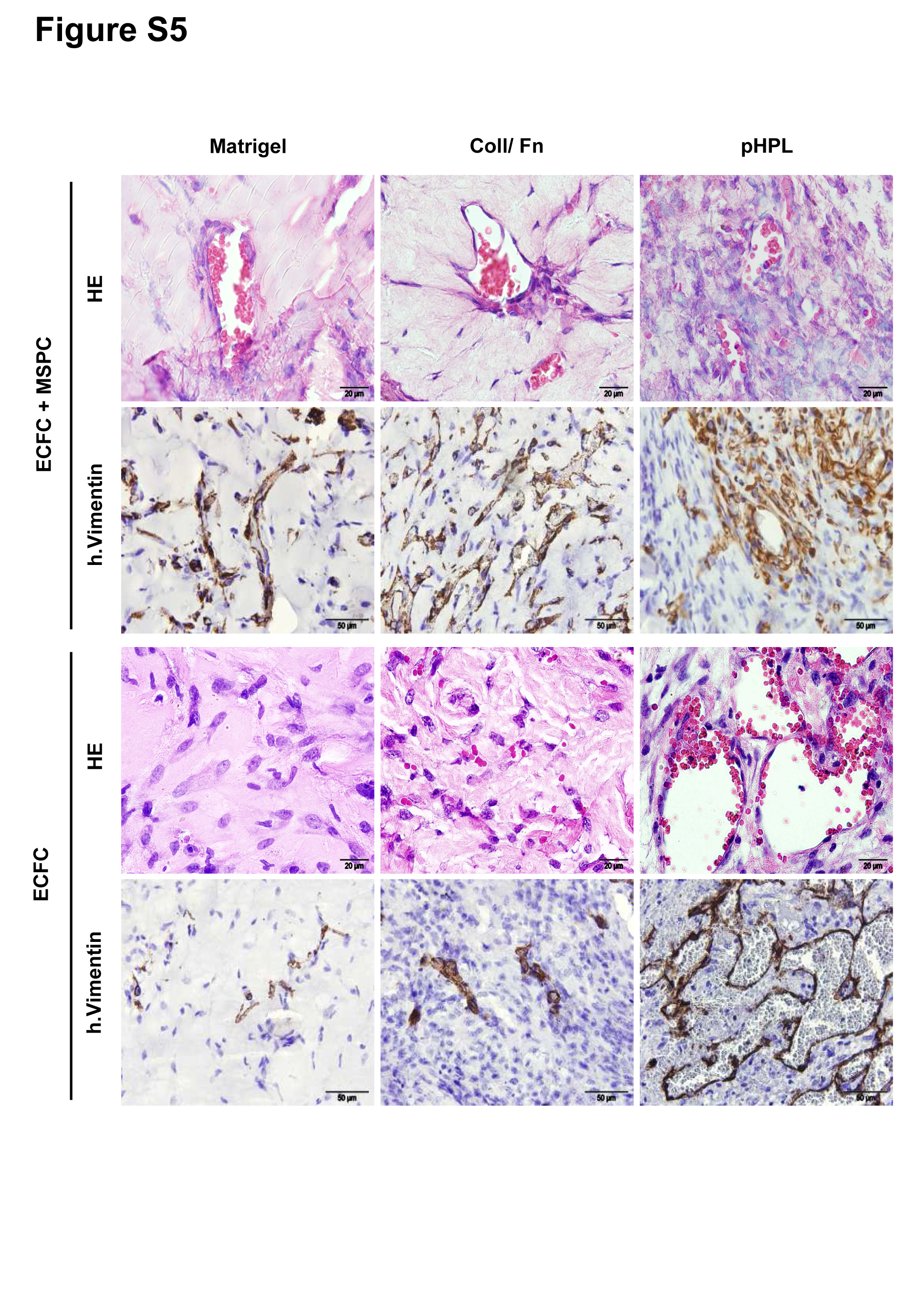

Supplement: Figure S5 — Patent vessel formation depends on functioning MSPCs and is virtually matrix- independent. ECFCs (1.6×10^6^) together with MSPCs (4×10^5^) for co-transplantation or sole ECFCs (2×10^6^) were re-suspended in ice cold matrigel, collagen/fibronectin (Coll/Fn), or pooled human platelet lysate (pHPL), respectively. Aliquots of matrigel or pHPL were injected and preformed collagen/fibronectin plugs were implanted subcutaneously as described in methods in detail into the flank of NSG mice. Mice were sacrificed on day (d) 7 and 1.5 µm plug sections were either stained with hematoxylin and eosin (HE) or processed for anti-human vimentin immune histochemistry (h.Vimentin; hematoxylin counterstain, blue; see methods for details and references). Control plugs were also explanted at d 1 (see Figure S4D). The higher cell density despite equal cell input in pHPL implants results from more intense contraction of the matrix in vivo compared to matrigel. (TIF) [file pone.0044468.s005.tif]

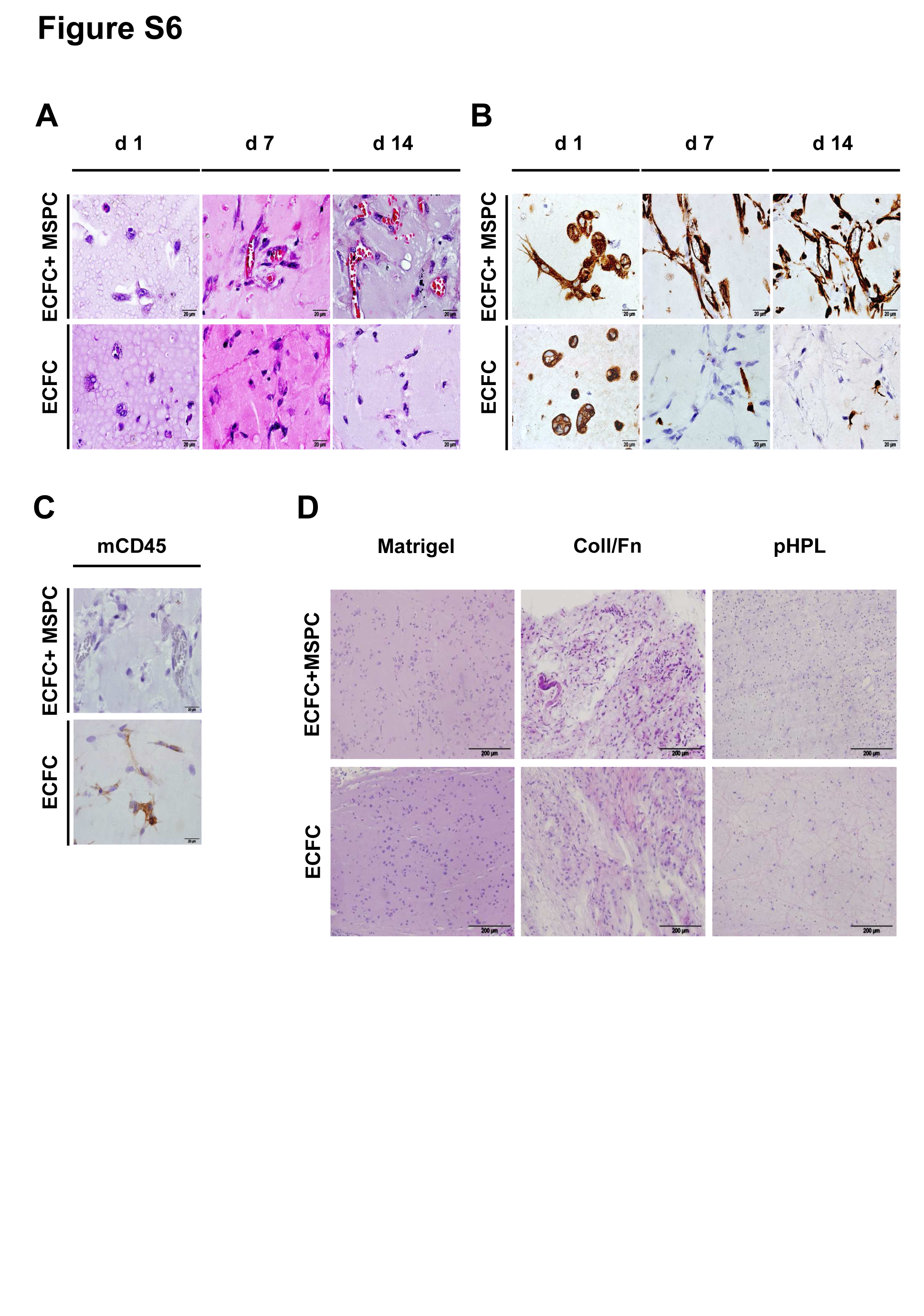

Supplement: Figure S6 — Patent vessel formation depends on MSPC presence and is virtually matrix-independent. (A, B) ECFCs alone or MSPCs+ECFCs (ratio 20∶80) were re-suspended in matrigel and injected subcutaneously into immune deficient NSG (NOD.Cg-Prkdcscid Il2rgtm1Wjl/SzJ) mice. Plugs where explanted at days (d) 1, 7 and 14. (A) Hematoxylin and eosin. (B) Mesodermal origin was probed with anti-human vimentin (brown; nuclei blue, hematoxylin). Implants after ECFC+MSPC co-transplantation showed vimentin+ human vessel formations (d7 & d14) compared to implants of ECFC alone showing not more than rare small vessel-like structures and declining human cell number. (C) Infiltrating vimentin-negative (non-human) cells in ECFC plugs were mouse CD45+ (mouse hematopoietic) cells already 7 d after transplantation. (D) Hematoxylin/eosin staining to visualize cells in different extracellular matrices 1 d after transplantation. MSPCs + ECFCs (top row) or ECFCs only (bottom row) were re-suspended in matrigel or pooled human platelet lysate (pHPL) and injected subcutaneously (6.6×10^6^/mL; ratio 20∶80; injection volume 300 µL) into immune-deficient NSG mice (NOD.Cg-Prkdcscid Il2rgtm1Wjl/SzJ; n ≥3). Preformed collagen/fibronectin plugs containing equal cell compositions were implanted (n = 3). (TIF) [file pone.0044468.s006.tif]

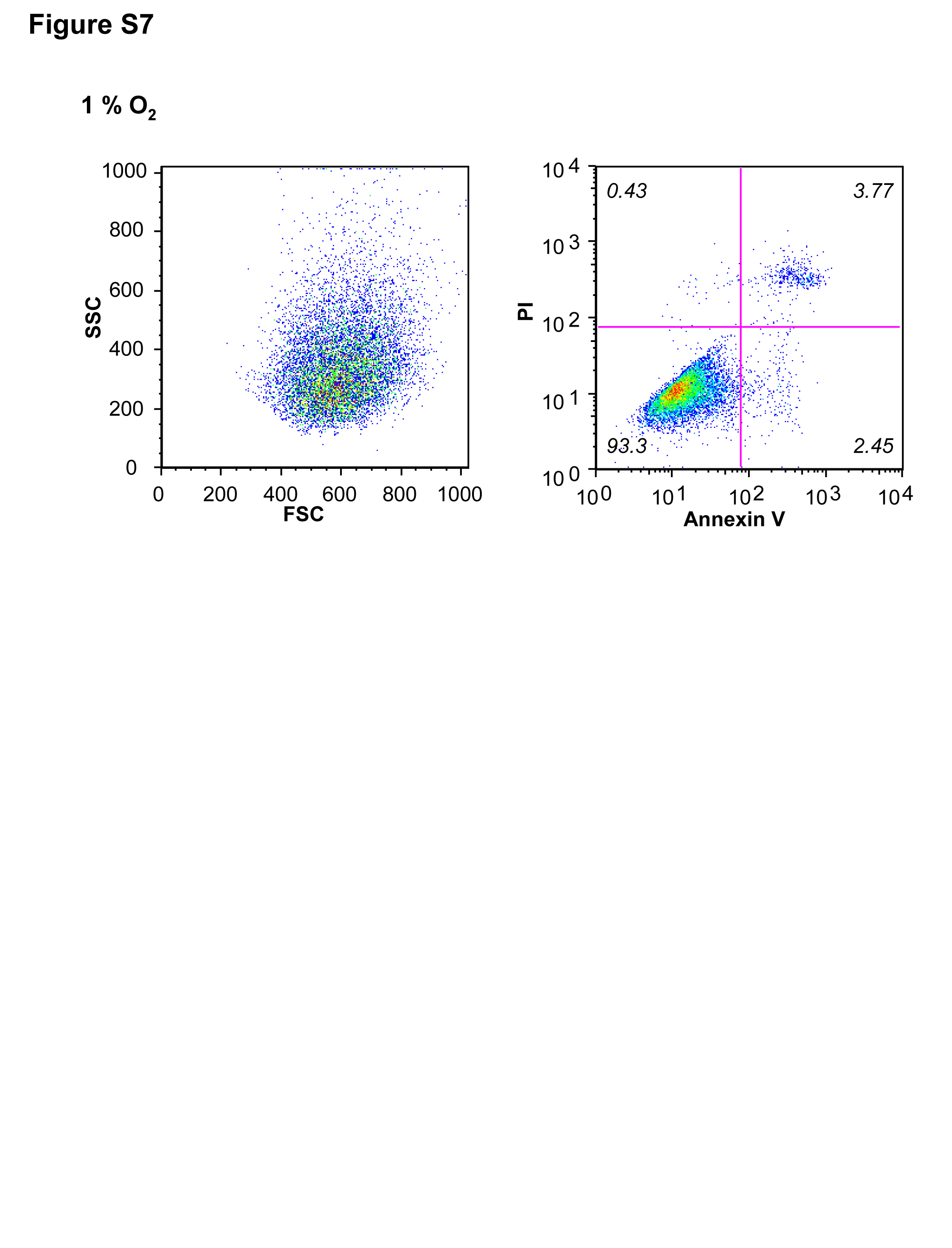

Supplement: Figure S7 — Anti-VEGF treatment inhibits vessel formation in vivo . (A) VEGF concentration in supernatant of MSPCs cultured for 3 days at 1%, 5% and 20% O2 showed increasing VEGF levels with decreasing oxygen concentration (mean ± SD; n = 3). (B) Anti-VEGF treatment inhibited vessel formation in matrigel plugs. After subcutaneous co-transplantation of MSPCs and ECFCs (ratio 20∶80) into NSG (NOD.Cg-Prdcscid Il2rgtm1Wjl/SzJ) mice recipients were injected i.p. with 5 mg/kg of the therapeutic anti-human VEGF antibody Bevacizumab every other day (d) starting d1. Hematoxylin/eosin staining showed limited cell arrangement but no human vessel formation after one week (d7; three doses of antibody). After seven doses of antibody (d14) some tiny vessels could be observed (arrows). Histology magnification is indicated by scale bar (200 µm). Macro-photography inserts show the freshly explanted pale plugs. (TIF) [file pone.0044468.s007.tif]

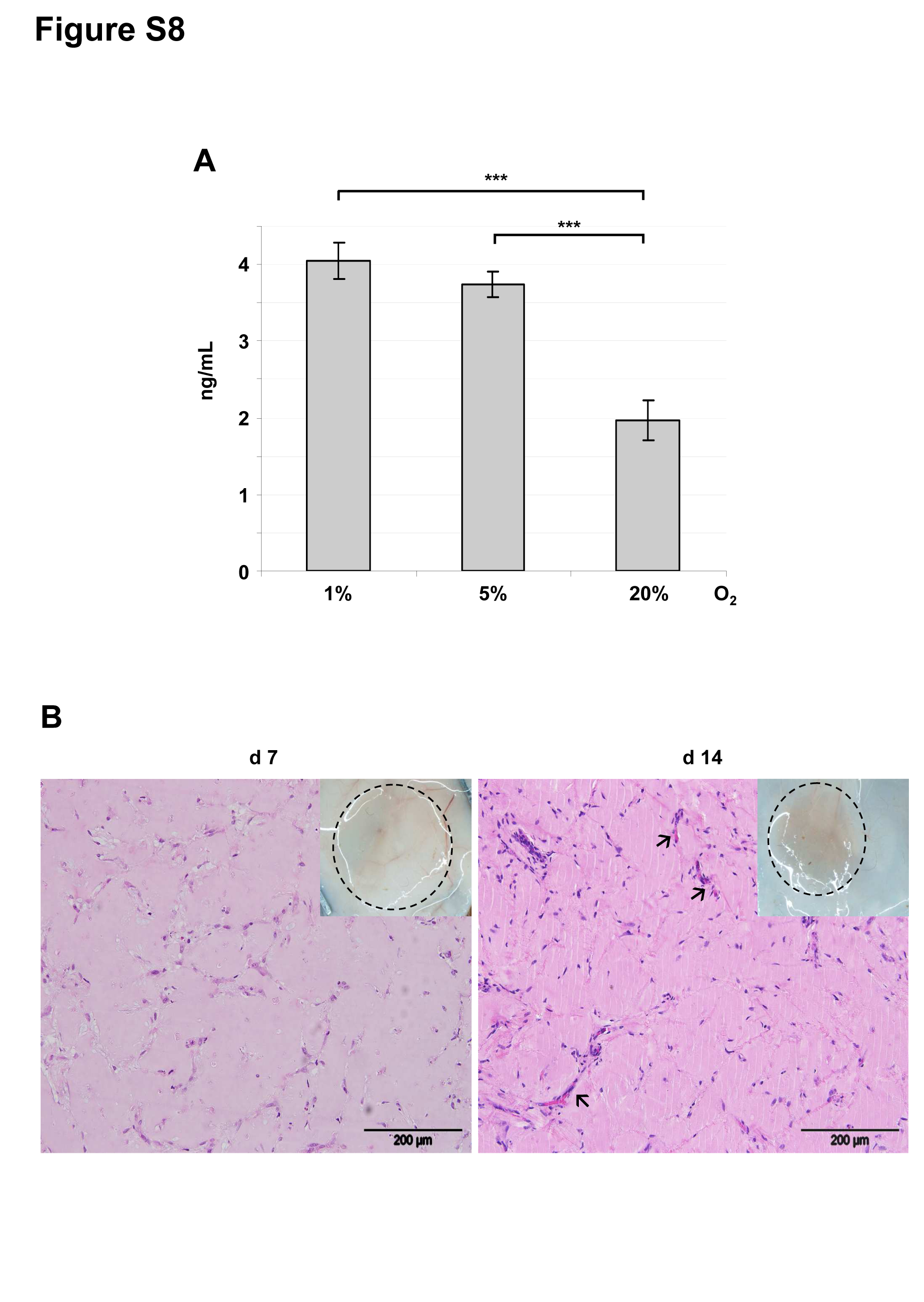

Supplement: Figure S8 — Apoptosis of MSPCs under hypoxia in vitro. Representative flow cytometry dot blot showing Annexin V binding to phosphatidylserine of apoptotic MSPCs combined with propidium iodide (PI) labeling after culture under hypoxic conditions (1% oxygen) for 8 days. Annexin V+/PI-(Annexin single positive) MSPCs represent apoptotic cells with an intact membrane excluding PI. Annexin V+/PI+ (double positive) MSPCs represent terminally dead cells which accumulate PI. (FSC, forward light scatter; SSC, side scatter). (TIF) [file pone.0044468.s008.tif]
